# Supplementary material for: Impact of repeat flooding on mental health and health-related quality of life: a cross-sectional analysis of the English National Study of Flooding and Health
Source: BMJ Open. 2019 Nov 2;9(11):e031562. doi: 10.1136/bmjopen-2019-031562 (PMC6830640; doi:10.1136/bmjopen-2019-031562)
Supplement: Supplementary data [file bmjopen-2019-031562supp001.pdf]

Appendix I. Socio-demographic characteristics of participants according to exposure to flooding

Characteristic	All participants (n=531)*	Exposure group		
		Unaffected (n=90)	Disrupted (n=322)	Flooded (n=119)
**Median age (IQR)**	62.4 (49.1 to 72.1)	64.0 (53.9 to 76.8)	61.9 (46.7 to 71.6)	61.3 (48.3 to 71.2)
**Sex**				
Male	217 (41.2%)	55 (61.1%)	113 (35.5%)	49 (41.2%)
Female	310 (58.8%)	35 (38.9%)	205 (64.5%)	70 (58.8%)
**Ethnicity**				
White	523 (99.6%)	88 (100%)	316 (99.4%)	119 (100%)
Other	2 (0.4%)	0 (0%)	2 (0.6%)	0 (0%)
**Marital Status**				
Married/civil partners/cohabiting	340 (64.6%)	68 (76.4%)	196 (61.6%)	76 (63.9%)
Separated/divorced/widowed	127 (24.1%)	16 (18.0%)	80 (25.2%)	31 (26.1%)
Single/other	59 (11.2%)	5 (5.6%)	42 (13.2%)	12 (10.1%)
**Housing tenure**				
Owner/joint owner/family owned	433 (82.3%)	73 (82.0%)	262 (82.4%)	98 (82.4%)
Other	93 (17.7%)	16 (18.0%)	56 (17.6%)	21 (17.6%)
**Employment**				
Employed	252 (47.8%)	36 (40.4%)	155 (48.4%)	61 (51.7%)
Retired	238 (45.2%)	47 (52.8%)	144 (45.0%)	47 (39.8%)
Other	37 (7.0%)	6 (6.7%)	21 (6.6%)	10 (8.5%)
**Education level**				
Degree or above	235 (44.8%)	29 (32.6%)	153 (48.2%)	53 (44.9%)
Below degree level	172 (32.8%)	32 (36.0%)	102 (32.2%)	38 (32.2%)
Other	46 (8.8%)	8 (9.0%)	29 (9.2%)	9 (7.6%)
None (no formal qualifications)	71 (13.5%)	20 (22.5%)	33 (10.4%)	18 (15.3%)
**English deprivation quintile**				
1 (least deprived)	49 (9.4%)	17 (19.1%)	30 (9.4%)	2 (1.7%)
2	221 (42.2%)	38 (42.7%)	138 (43.4%)	45 (38.5%)
3	202 (38.6%)	25 (28.1%)	114 (35.9%)	63 (53.9%)
4 and 5**	52 (9.9%)	9 (10.1%)	36 (11.3%)	7(6.0%)
**Long-term illness**				
Yes	394 (76.5%)	69 (80.2%)	241 (77.0%)	84 (72.4%)
No	121 (23.5%)	17(19.8%)	72 (23.0%)	32 (27.6%)

Note: proportions calculated among those with known information on the characteristic of interest
*With known exposure status
**The two most deprived quintiles were combined due to small numbers

Appendix II. EQ-5D-5L health-related dimensions according to exposure to flooding

EQ-5D-5L dimension	Level	Exposure to flooding					
		All	Unaffected	Disrupted	Flooded (all)	Single flooded	Repeat flooded
		n (%)	n (%)	n (%)	n (%)	n (%)	n (%)
**Mobility problems**	No	391 (75.2%)	65 (73.9%)	240 (76.4%)	86 (74.6%)	41 (75.9%)	45 (70.3%)
	Slight	59 (11.3%)	11 (12.5%)	36 (11.5%)	11 (9.3%)	1 (1.9%)	10 (15.6%)
	Moderate	49 (9.4%)	8 (9.1%)	25 (8.0%)	16 (13.6%)	10 (18.5%)	6 (9.4%)
	Severe/unable*	22 (4.2%)	4 (4.5%)	13 (4.1%)	5 (4.2%)	2 (3.7%)	3 (4.7%)
**Self-care problems**	No	482 (92.7%)	84 (95.5%)	290 (93.0%)	108 (91.5%)	49 (90.7%)	59 (92.2%)
	Slight	18 (3.5%)	2 (2.3%)	13 (4.1%)	3 (2.5%)	1 (1.9%)	2 (3.1%)
	Moderate	16 (3.1%)	2 (2.3%)	8 (2.5%)	6 (5.1%)	4 (7.4%)	2 (3.1%)
	Severe/unable*	4 (0.8%)	0 (0%)	3 (1.0%)	1 (0.8%)	0 (0%)	1 (1.6%)
**Activity problems**	No	403 (77.5%)	71 (80.7%)	247 (78.7%)	85 (72.0%)	37 (68.5%)	48 (75.0%)
	Slight	55 (10.6%)	9 (10.2%)	32 (10.2%)	14 (11.9%)	7 (13.0%)	7 (10.9%)
	Moderate	48 (9.2%)	7 (8.0%)	28 (8.9%)	13 (11.0%)	8 (14.8%)	5 (7.8%)
	Severe/unable*	14 (2.7%)	1 (1.1%)	7 (2.2%)	6 (5.1%)	2 (3.7%)	4 (6.3%)
**Pain/discomfort**	No	311 (59.8%)	51 (58.0%)	194 (61.8%)	66 (55.9%)	29 (53.7%)	37 (57.8%)
	Slight	122 (23.5%)	25 (28.4%)	67 (21.3%)	30 (25.4%)	15 (28.8%)	15 (23.4%)
	Moderate	63 (12.1%)	8 (9.1%)	39 (12.4%)	16 (13.6%)	7 (13.0%)	9 (14.1%)
	Severe/extreme*	24 (4.6%)	4 (4.5%)	14 (4.5%)	6 (5.1%)	3 (5.6%)	3 (4.7%)
**Anxiety/depression**	No	347 (66.7%)	71 (80.7%)	232 (73.9%)	44 (37.3%)	19 (35.2%)	25 (39.1%)
	Slight	96 (18.5%)	13 (14.8%)	53 (16.9%)	30 (25.4%)	18 (33.3%)	12 (18.8%)
	Moderate	60 (11.5%)	3 (3.4%)	23 (7.3%)	34 (28.8%)	14 (25.9%)	20 (31.3%)
	Severe/extreme*	17 (3.3%)	1 (1.1%)	6 (1.9%)	10 (8.5%)	3 (5.6%)	7 (10.9%)

*Groups combined due to small numbers
