# Supplementary material for: Impact of repeat flooding on mental health and health-related quality of life: a cross-sectional analysis of the English National Study of Flooding and Health
Source: BMJ Open. 2019 Nov 2;9(11):e031562. doi: 10.1136/bmjopen-2019-031562 (PMC6830640; doi:10.1136/bmjopen-2019-031562)
Supplement: Author's manuscript [file bmjopen-2019-031562.draft_revisions.pdf]

BMJ Open

BMJ Open is committed to open peer review. As part of this commitment we make the peer review history of every article we publish publicly available.

When an article is published we post the peer reviewers' comments and the authors' responses online. We also post the versions of the paper that were used during peer review. These are the versions that the peer review comments apply to.

The versions of the paper that follow are the versions that were submitted during the peer review process. They are not the versions of record or the final published versions. They should not be cited or distributed as the published version of this manuscript.

BMJ Open is an open access journal and the full, final, typeset and author-corrected version of record of the manuscript is available on our site with no access controls, subscription charges or pay-per-view fees (http://bmjopen.bmj.com).

If you have any questions on BMJ Open's open peer review process please email info.bmjopen@bmj.com

BMJ Open

Impact of repeat flooding on mental health and health-related quality of life: English National Study of Flooding and Health

Journal:	*BMJ Open*
Manuscript ID	bmjopen-2019-031562
Article Type:	Research
Date Submitted by the Author:	09-May-2019
Complete List of Authors:	French, Clare; University of Bristol, Population Health Sciences, Bristol Medical School<br>Waite, Thomas; Public Health England, National Infection Service<br>Armstrong, Ben; London School of Hygiene and Tropical Medicine<br>Rubin, GJ; King's College London, NIHR Health Protection Research Unit in Emergency Preparedness and Response, Department of Psychological Medicine, Weston Education Centre<br>National Study of Flooding and Health Study Group, English ; Public Health England<br>Beck, Charles; Public Health England, Field Service South West, National Infection Service; Bristol Medical School, NIHR Health Protection Unit in Evaluation of Interventions, Population Health Sciences<br>Oliver, Isabel; Public Health England, Field Service South West, National Infection Service; Bristol Medical School, NIHR Health Protection Unit in Evaluation of Interventions, Population Health Sciences
Keywords:	EPIDEMIOLOGY, PUBLIC HEALTH, MENTAL HEALTH

SCHOLARONE™
Manuscripts

Impact of repeat flooding on mental health and health-related quality of life: English National Study of Flooding and Health

Clare E French[1], Thomas D Waite[2], Ben Armstrong[3], G. James Rubin[4], English National Study of Flooding and Health Study Group, Charles R Beck[1,5] Isabel Oliver[1,5]

[1] NIHR Health Protection Unit in Evaluation of Interventions, Population Health Sciences, Bristol Medical School, University of Bristol, BS8 2PS, UK

[2] National Infection Service, Public Health England, London, UK

[3] NIHR Health Protection Research Unit in Environmental Change and Health at the London School of Hygiene and Tropical Medicine, London, UK

[4] Department of Psychological Medicine, Weston Education Centre, King's College London NIHR Health Protection Research Unit in Emergency Preparedness and Response, London, UK

[5] Field Service South West, National Infection Service, Public Health England, Bristol, UK

Corresponding author: Clare French, Population Health Sciences, Bristol Medical School, Canynge Hall, 39 Whatley Road, Bristol BS8 2PS, UK. Email: clare.french@bristol.ac.uk. Tel: +44(0)117 3313925

Word count: 3,056

For peer review only

For peer review only - http://bmjopen.bmj.com/site/about/guidelines.xhtml

ABSTRACT

Objective To assess the association between flooding/repeat flooding and: i) psychological morbidity (anxiety, depression, post-traumatic stress disorder [PTSD]) and ii) health-related quality of life (HRQoL) at six months post-flooding.

Design Cross-sectional analysis of data from the English National Study of Flooding and Health.

Setting Cumbria, England.

Participants Questionnaires were sent to 2500 residential addresses at six months post-flooding; 590 people responded.

Outcomes Probable depression was assessed using the Patient Health Questionnaire (PHQ-2); probable anxiety using the Generalised Anxiety Disorder scale (GAD-2); and probable PTSD using the short-form PTSD checklist (PCL-6). Health-related quality of life (HRQoL) was assessed using the EQ-5D-5L.

Mental health outcomes were analysed using logistic regression; HRQoL dimensions using ordinal regression; and summary index/VAS scores using linear regression.

Results One hundred and nineteen participants had been flooded, over half of whom were experiencing a repeat flooding event (54%; n=64).

Mental health outcomes were elevated among flooded compared with unaffected participants (adjusted odds ratio for probable depression: 7.77, 95%CI: 1.51 to 40.13; anxiety: 4.16, 95%CI: 1.18 to 14.70; PTSD: 14.41, 95%CI: 3.91 to 53.13). The prevalence of depression was higher among repeat compared with single flooded participants, but this was not significant after adjustment. There was no difference in levels of anxiety or PTSD.

Compared with unaffected participants, those flooded had lower EQ-5D-5L index scores (adjusted coefficient: -0.06, 95% CI: -0.12 to -0.01) and lower self-rated health scores (adjusted coefficient: -6.99, 95% CI: -11.96 to -2.02). There was, however, little difference in HRQoL overall between repeat and single flooded participants.

Conclusions Interventions are needed to help minimise the impact of flooding on people's mental health and HRQoL.

For peer review only

For peer review only - http://bmjopen.bmj.com/site/about/guidelines.xhtml

Strengths and limitations of this study

- There is currently very little research exploring the impact of repeat flooding on mental health or health-related quality of life – our research addresses a gap in the evidence
- We use data from the English National Study of Flooding and Health – a large, robust study with proven methodology.
- Three key mental health outcomes (depression, anxiety, post-traumatic stress disorder) as well as health-related quality of life were assessed, all using using validated instruments
- The tools used to assess psychological outcomes are indictive of probable diagnoses rather than being clinically diagnostic
- Information was not available on the number or timing previous flooding events

For peer review only

For peer review only - http://bmjopen.bmj.com/site/about/guidelines.xhtml

INTRODUCTION

Flooding can have a range of effects on people's health, both physical and psychological.[1-5] Given projections that both the frequency and severity of flooding events will continue to increase in the future,[6-9] populations living in flood-susceptible areas may be exposed to multiple flooding events. It is important that the impact of flooding, and repeat flooding, on people's health and wellbeing is thoroughly understood. This will help inform public health actions to mitigate adverse health consequences of future flooding events.

Beyond the acute health consequences of flooding, such as injuries and infections, the longer-term health consequences are thought to be primarily psychological.[6] The English National Study of Flooding and Health began in 2015 to assess the impact of widespread flooding in southern England. The study has assessed the impact of flooding and related disruption on mental health and wellbeing over several years. Data obtained one year after flooding showed higher levels of depression, anxiety and post-traumatic stress disorder (PTSD) in participants whose homes had been flooded, and to a lesser extent, those whose lives had been disrupted by flooding compared to those who were unaffected.[10] Follow-up of participants at two years post-flooding demonstrated that mental health consequences had waned but were still noticeably higher in affected groups.[11] The impact of flooding on health-related quality of life (HRQoL) has not yet been reported on.

Several factors are known to be associated with the likelihood of experiencing adverse mental health outcomes post-flooding. These include socio-demographic characteristics such as age, gender, socio-economic and education statuses, and factors related to the nature of flooding e.g. depth and duration.[12] Secondary stressors, including problems with relationships and loss of sentimental items have been associated with poor psychological outcomes.[13] Meanwhile, societal-level factors such as social cohesion and support, as well as individual-level factors such as positive/proactive coping strategies can be protective against the development of mental ill health in the aftermath of flooding.[2,14] Although there is less published research on the association between flooding and wellbeing/quality of life, the available evidence indicates that effects similarly occur through a complex set inter-related pathways.[15]

The UK Climate Change Risk Assessment 2017 highlighted flooding as a risk to people, communities and buildings that requires more action.[16] More research is required to assess and understand how these risks are best tackled. There is currently very limited research exploring the impact of experiencing repeated flooding events on mental health and/or HRQoL, and the evidence to-date is inconclusive.[14,17-19]

In early December 2015, exceptionally heavy and persistent rainfall associated with storm "Desmond", resulted in widespread flooding in Cumbria and other parts of northern Britain. Thousands of homes and businesses across Cumbria were flooded, and tens of thousands left without power.[20,21] Cumbria is an area that has been repeatedly affected by flooding - the region had experienced severe flooding just six years previously in 2009, and also in 2005.[21]

The objective of this study is to assess the association between flooding/repeat flooding and: i) psychological morbidity (probable anxiety, probable depression, probable PTSD) and ii) HRQoL, in the Cumbria region at six months post-flooding.

For peer review only - http://bmjopen.bmj.com/site/about/guidelines.xhtml

METHODS

Study conduct

Details on the design and conduct of the English National Study of Flooding and Health have been published previously.[10] Recruitment and data collection in Cumbria largely mirrored the original survey conducted elsewhere. Recruitment packs (including consent forms, study information sheets and questionnaires) were sent to 2500 residential households in postcode areas known to have been affected by the 2015/16 floods within four local authority areas (Allerdale, Carlisle, Eden, South Lakeland). The sample size was determined with the aim of sampling approximately 50% of flooded households. All adults in each household were invited to participate. Packs were sent out approximately six months post-flooding (May 2016), with two rounds of reminder letters sent to non-responders. Invitations were sent by post and participants had the option to respond either by post or online. Questionnaires were similar to those used for the original survey, with some minor clarification to the wording of questions, and the addition of the EQ-5D-5L instrument to assess HRQoL.

Participants were asked about their exposure to the 2015/16 floods, and whether they had been previously flooded. They were categorised as:

- Unaffected - those who experienced no flooding or disruption during the 2015/16 floods
- Disrupted - those who did not have floodwater in liveable areas of their homes but reported experiencing disruption due to the floods (e.g. floodwater in non-liveable areas such as a garage, disruption to loss of utilities, loss of access to services)
- Flooded - those who reported having floodwater in one or more liveable rooms of their home (e.g. living room, kitchen, bedroom). For analyses of repeat flooding this group was further sub-categorised into:
    - Repeat flooded - those who reported having been previously flooded (either in the same or a previous home),
    - Single flooded - those who reported not having been previously flooded.

Demographic information collected included date of birth, sex, ethnicity, long-term illness or disability, education, employment and marital statuses. Postcodes of residence were used to assign Lower Super Output Area deprivation scores based on the Index of Multiple Deprivation.[22] These were ranked according to scores for all of England and categorised into quintiles.

Mental health outcomes were assessed using validated instruments - probable depression using the Patient Health Questionnaire (PHQ-2) depression sub-scale; probable anxiety using the Generalised Anxiety Disorder scale (GAD-2); and probable PTSD using the short-form PTSD checklist (PCL-6). Cut-off scores, used to indicate the likely presence of the condition, were ≥3 for PHQ-2/GAD-2 and ≥14 for PCL-6.[23-25] We also generated a combined variable indicting the presence of 'any' of these probable conditions.

HRQoL was assessed using the EQ-5D-5L. This is a validated two-part instrument. The first part is a descriptive system assessing five health-related dimensions (mobility, self-care, usual activities, pain/discomfort and anxiety/depression) on a five-point scale (no problems, slight problems, moderate problems, severe problems and extreme problems). Information on these five health states was converted into a single index value. The second part is a visual analogue scale (VAS) which records the patient's self-rated health on a vertical scale from marked from 0

For peer review only - http://bmjopen.bmj.com/site/about/guidelines.xhtml

to 100 where the endpoints are 'The best health you can imagine' and 'The worst health you can imagine'.[26]

SelectSurvey (ClassApps, USA) was used to develop the online questionnaire and capture online responses. Data returned on paper forms were double-entered using EpiData (EpiData Association, Denmark) and cross-checked to identify any errors.

Data analysis

Data were analysed in STATA 13.1 (StataCorp, USA). Participants who did not provide enough information to enable an exposure status to be assigned to them were excluded from analyses by exposure group. For analyses of mental health outcomes, participants who did not complete an outcome instrument were excluded from analyses of that outcome only. HRQoL analyses were restricted to those with complete information on both parts of the tool.

Mental health outcomes were analysed using binary logistic regression. For analyses of HRQoL, the EQ-5D-5L descriptive system ordered categorical variables were analysed using ordinal logistic regression. The proportional odds assumption was checked for each outcome using approximate likelihood-ratio tests. Continuous variables (EQ-5D-5L index score and VAS) both had left-skewed distributions and are thus presented as medians and inter-quartile ranges (IQRs). Medians were compared using the Mann Whitney U test and adjusted analyses were conducted using linear regression with robust standard errors to allow for the skewed distribution of the dependent variable.

Potential socio-demographic confounders of the association between flooding and mental health outcomes have been previously identified (age group, sex, local authority, ethnicity, marital status, education level, employment, deprivation score and pre-existing illness).[10] All (except ethnicity, due to very small numbers in the non-white ethnic group) were adjusted for *a priori* in all multivariable analyses for consistency and comparability between models.

Patient and public involvement

A community flooding action group was involved in the design of the study. Volunteers identified by the National Flood Forum piloted early versions of the questionnaires, and the National Flood Forum Chief Executive is a member of our stakeholder group. Participants were invited to join a study group to support the project and keep track of its progress. Study findings are being disseminated to participants via reports published online.

RESULTS

Response rates

Completed questionnaires were received from 590 participants (household response rate: 24.4%). More than one individual responded from 19 households giving a total of 628 participating individuals. Most participants responded using the paper questionnaire, with 7.0% (n=44) responding online. Subsequent analyses were conducted on the 531 participants who provided sufficient information to enable an exposure status to be assigned to them.

Participant characteristics

For peer review only - http://bmjopen.bmj.com/site/about/guidelines.xhtml

The socio-demographic characteristics of study participants are provided in Appendix I. Overall, the median age was 69 years (inter-quartile range [IQR]: 49 to 72 years), 58.8% were female, 99.6% were of white ethnicity, 51.6% lived in areas belonging to the two least deprived quintiles and 76.5% had a long-term illness or disability.

Exposure to flooding and psychological morbidity

Overall, 16.9% of participants were unaffected by the floods, 60.6% were disrupted and 22.4% were flooded.

After adjusting for socio-demographic factors, there was an elevated odds of each mental health outcome among flooded participants compared to those unaffected (adjusted odds ratio [aOR] for probable depression: 7.77, 95% confidence interval [CI]: 1.51 to 40.13; anxiety: 4.16, 95% CI: 1.18 to 14.70; PTSD: 14.41, 95% CI: 3.91 to 53.13; and for 'any' of these mental health outcomes: 11.81, 95% CI: 3.99-34.96). The adjusted odds of probable depression, PTSD and any outcome (but not anxiety) were also somewhat raised for those who experienced disruption due to flooding but not significantly so (Table 1).

Of those flooded during the 2015/16 floods, over half (54%; 64/119) had been previously flooded (60 at the same home and four at a previous home). The prevalence of probable depression was elevated among those exposed to repeated, as compared with a single flooding event, but the difference reduced on adjustment for potential confounders, and was not significant. For probable anxiety and PTSD there was little difference in prevalence among people who experienced single and repeat flooding, either before or after adjustment (Table 1).

Exposure to flooding and HRQoL

Most participants (97.9%, n=520) had complete information on both parts of the EQ-5D-5L.

Index scores

Median EQ-5D-5L index scores are presented in Table 2. In adjusted analyses flooded participants (all) had lower EQ-5D-5L index scores than unaffected participants (adjusted coefficient:-0.06, 95% CI:-0.12 to -0.01). There was little evidence that scores differed between repeat and single flooded participants.

Health-related dimensions

The level of problems reported by participants on each dimension, according to exposure to flooding are provided in Appendix II.

Flooded participants were more likely to have higher anxiety/depression scores than those unaffected (aOR: 8.59, 95% CI:4.02 to 18.36; Table 3), also more self-care and activity problems, though not significantly so. There was little evidence that the flooded group had more mobility problems or pain/discomfort than those unaffected. There was little evidence of a difference between repeat and single flooded participants on any dimension (Table 3). There was no evidence against the proportional odds assumption for any of the five dimensions.

VAS scores

Median VAS scores are presented in Table 4 – scores were 78 (IQR 69 to 90) for flooded participants, notably lower than for those unaffected (90, IQR: 75 to 93). This difference remained after adjustment (adjusted coefficient of VAS scores among flooded vs. unaffected participants: -6.99, 95% CI: -11.96 to -2.02). There was little evidence of a difference in VAS

For peer review only - http://bmjopen.bmj.com/site/about/guidelines.xhtml

scores between the repeat and single flooded groups. For analyses of both index scores and VAS scores the key assumptions of linear regression were met; robust standard errors were used which dealt with the non-normality of the data.

DISCUSSION

This study provides strong evidence of an association between flooding, be it a single or repeat episode, and probable mental health outcomes. Flooded participants also tended to have poorer HRQoL than those unaffected by the floods. Analyses of the dimension-specific descriptive EQ-5D-5L system indicate that anxiety/depression may be an important driver of this association. There was little evidence of a difference in either probable mental health outcomes or HRQoL between repeat and single flooded participants, although data were suggestive of a greater level of depression in those repeatedly flooded.

The UK Climate Change Risk Assessment 2017 identifies flooding as a major risk to people and the built environment; more action is needed to mitigate this risk.[16] The UK government has committed to actions, such as ensuring that people have good access to information to assess the risks, including health risks, of flooding; helping people to make their properties more resistant to flooding; and ensuring plans are in place to predict and respond to flooding incidents.[27] Our research should be used to inform that government action - although we focus on the impact of flooding on health and wellbeing, the evidence presented here supports the case for good town planning, flood and coastal risk management, and the implementation of the 25-year Environment Plan.[27]

The overall associations between being flooded (vs. unaffected) and mental health outcomes were consistent with those measured in the cohort affected by the floods in the winter of 2013/14 in Southern England.[10] Those floods largely affected affluent areas potentially limiting the generalisability of the findings beyond that population. Participants from Cumbria resided in areas of comparatively greater deprivation; our findings thus demonstrate that the increased risks of mental health outcomes following flooding are not restricted to those living in affluent areas. This is consistent with low income having been previously reported as a known driver of vulnerability to the effects of flooding.[28] Of note, a strong association between flooding and anxiety/depression was found using the EQ-5D-5L, corroborating findings obtained using mental health outcome assessment tools.

There is limited previous research on the impact of flooding on HRQoL with which to draw comparisons, and we are not aware of any previously published comparable data on the impact of repeat flooding specifically. A mixed-methods study in two UK regions found that flooding had a marked impact on wellbeing. This occurred via a complex and diverse range of interacting factors acting at both the individual and societal level.[15] Data from China showed poorer quality of life among those affected by floods compared with those unaffected, but the EQ-5D-5L tool was not used and the findings are not directly comparable.[29]

That over half of flooded participants in our sample had experienced repeat flooding is striking. It may be hypothesized that this group will suffer greater mental health and HRQoL effects than those experiencing single event due to the cumulative effects of these experiences, but our research indicates that any association may be more nuanced. The data were suggestive of higher levels of depression, but not other mental health outcomes or overall HRQoL, among repeat compared with single flooded participants. However, the number of events were small in

For peer review only - http://bmjopen.bmj.com/site/about/guidelines.xhtml

some groups resulting in imprecise estimates – the data should thus be interpreted with some caution.

Repeat flooding may impact on mental health in a variety of ways, though the evidence is inconclusive.[14,17,18] At the individual level, experiencing a repeat episode of flooding may trigger memories of previous flooding episodes, intensifying the distress experienced.[17] Those who have been previously flooded may also experience anticipatory anxiety and stress about the prospect of future flooding.[30,31] Conversely, people's individual and collective coping capacity may be increased, and they may display greater resilience to future flooding events.[32] Factors acting at the community level may mitigate the risks of adverse consequences of future floods through the development or strengthening of community networks and increased social cohesion.[15,30,33] Past flood events may have also resulted in improvements to infrastructure, flood defences and response systems, reducing the extent of subsequent floods and impact on people's lives.

General limitations of the National Study of Flooding and Health have been outlined elsewhere.[10] A limitation of these analyses specifically is that we do not have any information on the number, timing or extent of previous flooding experiences. Such information would be helpful in interpreting our findings.

Though validated instruments, the tools used to assess psychological outcomes are not clinically diagnostic. Our data therefore indicate 'probable' diagnoses which are likely to overestimate the number with a diagnosable mental health condition. Meanwhile, the EQ-5D-5L is a validated and widely used tool that has been previously used in UK populations.[34] The tool was well completed (98% of participants had complete data) indicating its acceptability in our study. However, some of the dimensions assessed may not be directly relevant to the types of illnesses that may occur as a consequence of flooding. At the same time, due to space constraints on the questionnaire, we were unable to assess all psychological variables that are of theoretical interest following repeat flooding. For example, anger might be elevated in flooded, and especially repeat flooded, participants and may be worthy of future investigation.

Our findings may not be generalisable to all populations affected by flooding (e.g. there were few participants of non-white ethnicity). However, all comparison groups were drawn from the same population and key socio-demographic confounding factors were adjusted for in the analyses.

Conclusions and recommendations

Flooding can have a profound effect on people's psychological health, and aspects of their HRQoL. Though we found little evidence that people exposed to repeat flooding were at increased risk of adverse outcomes compared with those exposed to a single event, it should be remembered that these individuals are at risk of experiencing adverse outcomes each time they experience flooding.

That over half of participants in this study were experiencing a repeat flooding event highlights the need for strengthening flood defences and mitigating risks of future flooding in areas at risk or previously affected. The UK's National Adaptation Programme for climate change recommends actions for reducing flood risk and harm from flooding;[27] the evaluation of these planned actions should include an assessment of their impact on people's mental health and wellbeing.

Interventions are needed to help minimise the impact of flooding on people's mental health and their wellbeing. Early warning systems can reduce the effects of flooding on mental health.[35] Providing people with other approaches to reduce their susceptibility to the effects of flooding,

For peer review only - http://bmjopen.bmj.com/site/about/guidelines.xhtml

For peer review only

such as efforts to increase community cohesion and develop support networks in preparedness for potential future flooding events, may also be beneficial. Finally, ensuring that adequate and effective mental health services are available for community members following flooding is vital.

For peer review only

For peer review only - http://bmjopen.bmj.com/site/about/guidelines.xhtml

Acknowledgments
We would like to thank the participants in this study and are grateful for the support we received from Colin Cox, Director of Public Health Cumbria County Council and the North West Public Health England Centre. We acknowledge the role of other members of the English National Study of Flooding and Health Study Group in the design of the National Study of Flooding and Health: Katerina Chaintarli, Angie Bone, Richard Amlôt, Sari Kovats, Mark Reacher and Giovanni Leonardi. We would also like to thank all PHE staff who contributed to study administration, set up and data entry.

Part of this work was presented at the Public Heath England Public Health Research and Science Conference 2018, Warwick, UK.

Ethics approval
Ethical approval for the study was granted by the Psychiatry, Nursing and Midwifery Research Ethics Subcommittee at King's College London [Reference PNM 1314 152]. All participants consented to participate in the study.

Data sharing
The datasets analysed in this study are available from Public Health England Field Epidemiology Service on reasonable request.

Competing interests
The authors declare that they have no competing interests.

Funding
The research was funded in part by the National Institute for Health Research Health Protection Research Units (NIHR HPRU) in Emergency Preparedness and Response at King's College London, Environmental Change at the London School of Hygiene and Tropical Medicine and Evaluation of Interventions at the University of Bristol, in partnership with Public Health England (PHE). The views expressed are those of the authors and not those of the NHS, the NIHR, The Department of Health or Public Health England.

Author contributions
CEF led the design and conduct of these analyses, with expert statistical advice from BA, and drafted the manuscript. English National Study of Flooding and Health Study Group members including named authors IO, TDW, BA, CRB and GJR designed the National Study of Flooding and Health and acquired the data. TDW, BA, CRB, GJR, and IO contributed to the design of the analyses and the interpretation the findings. All authors contributed to the writing of the manuscript and approved it for submission.

Exclusive license
The Submitting Author accepts and understands that any supply made under these terms is made by BMJ to the Submitting Author unless you are acting as an employee on behalf of your employer or a postgraduate student of an affiliated institution which is paying any applicable article publishing charge ("APC") for Open Access articles. Where the Submitting Author wishes to make the Work available on an Open Access basis (and intends to pay the relevant APC), the terms of reuse of such Open Access shall be governed by a Creative Commons licence – details of these licences and which Creative Commons licence will apply to this Work are set out in our licence referred to above.

For peer review only - http://bmjopen.bmj.com/site/about/guidelines.xhtml

Table 1. Prevalence and odds ratios (crude and adjusted) of mental health outcomes according to exposure to flooding

	n/N (%)*	OR (95% CI)	aOR** † (95% CI)
**MODEL 1 - Comparing flooded and disrupted with unaffected participants**			
**Probable depression**			
Unaffected	2/83 (2.4%)	Ref	Ref
Disrupted	15/292 (5.1%)	2.19 (0.49 to 9.80)	1.38 (0.27 to 6.96)
Flooded	25/106 (23.6%)	12.50 (2.87 to 54.52)	7.77 (1.51 to 40.13)
**Probable anxiety**			
Unaffected	5/83 (6.0%)	Ref	Ref
Disrupted	19/289 (6.6%)	1.10 (0.40 to 3.03)	0.91 (0.27 to 3.08)
Flooded	24/107 (22.4%)	4.51 (1.64 to 12.41)	4.16 (1.18 to 14.70)
**Probable PTSD**			
Unaffected	3/87 (3.4%)	Ref	Ref
Disrupted	30/300 (10.0%)	3.11 (0.93 to 10.45)	2.05 (0.57 to 7.40)
Flooded	49/112 (43.8%)	21.78 (6.49 to 73.08)	14.41 (3.91 to 53.13)
**'Any' probable mental health outcome**			
Unaffected	6/82 (7.3%)	Ref	Ref
Disrupted	41/285 (14.4%)	2.13 (0.87 to 5.21)	1.88 (0.67 to 5.32)
Flooded	52/107 (48.6%)	11.98 (4.08 to 29.86)	11.81 (3.99 to 34.96)
**MODEL 2 - Comparing repeat with single flooded participants**			
**Probable depression**			
Single flooded	8/49 (16.3%)	Ref	Ref
Repeat flooded	17/57 (29.8%)	2.18 (0.85 to 5.61)	1.55 (0.44 to 5.46)
**Probable anxiety**			
Single flooded	11/50 (22.0%)	Ref	Ref
Repeat flooded	13/57 (22.8%)	1.05 (0.42 to 2.61)	0.89 (0.26 to 2.97)
**Probable PTSD**			
Single flooded	21/51 (41.2%)	Ref	Ref
Repeat flooded	28/61 (45.9%)	1.21 (0.57 to 2.57)	0.73 (0.27 to 1.97)
**'Any' probable mental health outcome**			
Single flooded	22/48 (45.8%)	Ref	Ref
Repeat flooded	30/59 (50.8%)	1.22 (0.57 to 2.62)	1.03 (0.39 to 2.73)

OR – odds ratio, aOR – adjusted odds ratio, CI – confidence interval

*N is the total number of participants in each exposure group for whom a complete answer to this outcome instrument was provided, and % is the proportion of those who have the relevant (probable) condition

For peer review only - http://bmjopen.bmj.com/site/about/guidelines.xhtml

**Adjusted for age group, sex, local authority, marital status, education level, employment, deprivation score and pre-existing illness

†Number of observations included in the multivariable models were: probable depression n=446, probable anxiety n=444, probable PTSD n=464, 'any' probable mental health outcome n=440. Both models included the same participants but the reference group differs and odds ratios are only presented for the comparison of interest

For peer review only

For peer review only - http://bmjopen.bmj.com/site/about/guidelines.xhtml

Table 2. Univariable and multivariable analyses of the association between exposure to flooding and EQ-5D-5L index values

	Exposure group	Median (IQR) index value	Crude coefficient (95% CI)*	Adjusted coefficient (95% CI)* **
MODEL 1	Unaffected	1.00 (0.77 to 1.00)	Ref	Ref
	Disrupted	1.00 (0.77 to 1.00)	-0.00 (-0.52 to 0.04)	-0.00 (-0.44 to 0.04)
	Flooded	0.85 (0.69 to 1.00)	-0.08 (-0.14 to -0.02)	-0.06 (-0.12 to -0.01)
MODEL 2	Single flooded	0.84 (0.68 to 0.88)	Ref	Ref
	Repeat flooded	0.85 (0.69 to 1.00)	0.00 (-0.08 to 0.08)	0.02 (-0.05 to 0.09)

*Confidence intervals estimated using robust standard errors

**Adjusted for age group, sex, local authority, marital status, education level, employment, deprivation score and pre-existing illness

For peer review only

For peer review only - http://bmjopen.bmj.com/site/about/guidelines.xhtml

Table 3. Univariable and multivariable analyses of EQ-5D-5L health-related dimensions according to exposure to flooding

	OR (95% CI)	aOR (95% CI)* **
**MODEL 1 - Comparing flooded and disrupted with unaffected participants**		
**Mobility problems**		
Unaffected	Ref	Ref
Disrupted	0.87 (0.51 to 1.49)	0.98 (0.48 to 2.02)
Flooded	1.09 (0.59 to 2.02)	1.04 (0.44 to 2.46)
**Self-care problems†**		
Unaffected	Ref	Ref
Disrupted	1.73 (0.58 to 5.12)	1.61 (0.41 to 6.31)
Flooded	1.98 (0.60 to 6.52)	1.62 (0.33 to 7.91)
**Activity problems**		
Unaffected	Ref	Ref
Disrupted	1.15 (0.64 to 2.07)	1.59 (0.70 to 3.60)
Flooded	1.68 (0.87 to 3.25)	1.85 (0.72 to 4.79)
**Pain/discomfort**		
Unaffected	Ref	Ref
Disrupted	0.92 (0.58 to 1.46)	0.95 (0.54 to 1.68)
Flooded	1.14 (0.67 to 1.95)	1.01 (0.50 to 2.02)
**Anxiety/depression**		
Unaffected	Ref	Ref
Disrupted	1.51 (0.84 to 2.70)	1.46 (0.73 to 2.91)
Flooded	7.72 (4.11 to 14.50)	8.59 (4.02 to 18.36)
**MODEL 2 - Comparing repeat with single flooded participants**		
**Mobility problems**		
Single flooded	Ref	Ref
Repeat flooded	1.16 (0.52 to 2.63)	0.85 (0.28 to 2.54)
**Self-care problems†**		
Single flooded	Ref	Ref
Repeat flooded	0.82 (0.23 to 3.01)	0.42 (0.08 to 2.29)
**Activity problems**		
Single flooded	Ref	Ref
Repeat flooded	0.74 (0.33 to 1.64)	0.71 (0.24 to 2.11)
**Pain/discomfort**		
Single flooded	Ref	Ref
Repeat flooded	0.88 (0.43 to 1.77)	0.52 (0.21 to 1.26)
**Anxiety/depression**		
Single flooded	Ref	Ref
Repeat flooded	1.20 (0.62 to 2.32)	1.13 (0.51 to 2.51)

For peer review only

For peer review only - http://bmjopen.bmj.com/site/about/guidelines.xhtml

*Adjusted for age group, sex, local authority, marital status, education level, employment, deprivation score and pre-existing illness

**Number of observations included in all multivariable models was 484. Both models included the same participants, but the reference group differed and odds ratios are only presented for the comparison of interest

†Due to very small numbers of participants with ‘severe/extreme problems’ on the self-care dimension, the ‘severe/extreme’ and ‘moderate’ categories were combined

For peer review only

Table 4. Univariable and multivariable analyses of the association between exposure to flooding and EQ-5D-5L VAS scores

	Exposure group	Median (IQR) VAS score	Crude coefficient (95% CI)*	Adjusted coefficient (95% CI)* **
MODEL 1	Unaffected	90 (75 to 93)	Ref	Ref
	Disrupted	85 (75 to 90)	-2.18 (-6.15 to 1.79)	-1.77 (-5.45 to 1.91)
	Flooded	78 (69 to 90)	-8.66 (-13.66 to -3.67)	-6.99 (-11.96 to -2.02)
MODEL 2	Single flooded	75 (70 to 85)	Ref	Ref
	Repeat flooded	80 (65 to 90)	-0.64 (-7.78 to 6.50)	0.76 (-7.06 to 8.60)

*Confidence intervals estimated using robust standard errors

**Adjusted for age group, sex, local authority, marital status, education level, employment, deprivation score and pre-existing illness

For peer review only

References

1. Ahern M, Kovats RS, Wilkinson P, et al. Global health impacts of floods: epidemiologic evidence. *Epidemiol Rev* 2005;27:36-46.

2. Fernandez A, Black J, Jones M, et al. Flooding and mental health: a systematic mapping review. *PLoS One* 2015;10(4):e0119929.

3. Saulnier DD, Brolin Ribacke K, von Schreeb J. No Calm After the Storm: A Systematic Review of Human Health Following Flood and Storm Disasters. *Prehosp Disaster Med* 2017;32(5):568-79.

4. Stanke C, Murray V, Amlot R, et al. The effects of flooding on mental health: Outcomes and recommendations from a review of the literature. *PLoS Currents* 2012; e4f9f1fa9c3cae. doi:10.1371/4f9f1fa9c3cae.

5. Zhong S, Yang L, Toloo S, et al. The long-term physical and psychological health impacts of flooding: A systematic mapping. *Sci Total Environ* 2018;626:165-94.

6. World Health Organization Regional Office for Europe. Floods in the WHO European Union: health effects and their prevention. Copenhagen, Denmark: World Health Organization, 2013:1-146. http://www.euro.who.int/__data/assets/pdf_file/0020/189020/e96853.pdf (Accessed September 2018).

7. European Environment Agency, European Commission Joint Research Centre and the World Health Organization. Impacts of Europe's changing climate - 2008 indicator-based assessment. Joint EEA-JRC-WHO report. Copenhagen, Denmark: European Environment Agency, European Commission Joint Research Centre and the World Health Organization, 2008:1-246. https://ec.europa.eu/jrc/sites/jrcsh/files/jrc_reference_report_2008_09_climate_change.pdf (Accessed September 2018).

8. Alfieri L, Bisselink B, Dottori F, et al. Global projections of river flood risk in a warmer world. *Earths Future* 2017;5(2):171-82.

9. Sayers PB, Horritt M, Penning-Rowsell E, et al. Climate Change Risk Assessment 2017: Projections of future flood risk in the UK. London, UK: Committee on Climate Change, 2017. https://www.theccc.org.uk/wp-content/uploads/2015/10/CCRA-Future-Flooding-Main-Report-Final-06Oct2015.pdf (Accessed October 2018).

10. Waite TD, Chaintarli K, Beck CR, et al. The English national cohort study of flooding and health: cross-sectional analysis of mental health outcomes at year one. *BMC Public Health* 2017;17(1):129.

11. Jermacane D, Waite TD, Beck CR, et al. The English National Cohort Study of Flooding and Health: the change in the prevalence of psychological morbidity at year two. *BMC Public Health* 2018;18(1):330.

12. Lowe D, Ebi KL, Forsberg B. Factors increasing vulnerability to health effects before, during and after floods. *Int J Environ Res Public Health* 2013;10(12):7015-67.

13. Tempest EL, English National Study on F, Health Study G, et al. Secondary stressors are associated with probable psychological morbidity after flooding: a cross-sectional analysis. *Eur J Public Health* 2017;27(6):1042-47.

14. Greene G, Paranjothy S, Palmer SR. Resilience and Vulnerability to the Psychological Harm From Flooding: The Role of Social Cohesion. *Am J Public Health* 2015;105(9):1792-5.

15. Walker-Springett K, Butler C, Adger WN. Wellbeing in the aftermath of floods. *Health Place* 2017;43:66-74.

For peer review only - http://bmjopen.bmj.com/site/about/guidelines.xhtml

16. Committee on Climate Change. UK Climate Change Risk Assessment 2017. Synthesis report: priorities for the next five years. London, UK: Committee on Climate Change, 2016. https://www.theccc.org.uk/wp-content/uploads/2016/07/UK-CCRA-2017-Synthesis-Report-Committee-on-Climate-Change.pdf (Accessed October 2018).

17. Lamond J. The role of flood memory in the impact of repeat flooding on mental health. In: Proverbs D. and Brebbia, C., eds. *Flood Recovery, Innovation and Response IV*. Ashurst, UK: WIT Press, 2014. https://www.witpress.com/books/978-1-84564-784-1 (Accessed August 2018)

18. Mason V, Andrews H, Upton D. The psychological impact of exposure to floods. *Psychol Health Med* 2010;15(1):61-73.

19. Norris FH, Murrell SA. Prior experience as a moderator of disaster impact on anxiety symptoms in older adults. *Am J Community Psychol* 1988;16(5):665-83.

20. Met Office. Flooding in Cumbria December 2015. Secondary Flooding in Cumbria December 2015. https://www.metoffice.gov.uk/climate/uk/interesting/december2015. (Accessed July 2018).

21. Miller JD, Kjeldsen TR, Hannaford J, et al. A hydrological assessment of the November 2009 floods in Cumbria, UK. *Hydrology Research* 2013;44(1):180-97.

22. Department for Communities and Local Government. English indices of deprivation 2015. Secondary English indices of deprivation 2015. https://www.gov.uk/government/statistics/english-indices-of-deprivation-2015 (Accessed May 2018).

23. Kroenke K, Spitzer RL, Williams JB. The Patient Health Questionnaire-2: validity of a two-item depression screener. *Med Care* 2003;41(11):1284-92.

24. Kroenke K, Spitzer RL, Williams JB, et al. Anxiety disorders in primary care: prevalence, impairment, comorbidity, and detection. *Ann Intern Med* 2007;146(5):317-25.

25. Lang A, Stein M. An abbreviated PTSD checklist for use as a screening instrument in primary care. *Behaviour Research and Therapy* 2005;43(5):585-94.

26. van Reenen M, Janssen B. EQ-5D-5L User Guide. Version 2.1,2015:1-28. https://euroqol.org/wp-content/uploads/2016/09/EQ-5D-5L_UserGuide_2015.pdf (Accessed June 2018)

27. Department for Environment, Food and Rural Affairs. The National Adaptation Programme and the Third Strategy for Climate Adaptation Reporting. London, UK: Department for Environment, 2018. https://assets.publishing.service.gov.uk/government/uploads/system/uploads/attachment_data/file/727252/national-adaptation-programme-2018.pdf (Accessed September 2018).

28. Sayers PB, Horritt M, Penning Rowsell E, et al. Present and future flood vulnerability, risk and disadvantage: A UK scale assessment. A report for the Joseph Rowntree Foundation. Watlington, UK: Sayers and Partners LLP, 2017:1-98. http://www.sayersandpartners.co.uk/uploads/6/2/0/9/6209349/sayers_2017__present_and_future_flood_vulnerability_risk_and_disadvantage_-_final_report_-_uploaded_05june2017_printed_-_high_quality.pdf (Accessed September 2018).

29. Tan HZ, Luo YJ, Wen SW, et al. The effect of a disastrous flood on the quality of life in Dongting lake area in China. *Asia Pac J Public Health* 2004;16(2):126-32.

30. Tapsell SM, Penning-Rowsell EC, Tunstall SM, et al. Vulnerability to flooding: health and social dimensions. *Philos Trans A Math Phys Eng Sci* 2002;360(1796):1511-25.

For peer review only - http://bmjopen.bmj.com/site/about/guidelines.xhtml

31. Tunstall S, Tapsell S, Green C, et al. The health effects of flooding: Social research results from England and Wales. *J Water Health* 2006;4(3):365-80.

32. Bulter C, Walker-Springett K, Adger WN, et al. Social and Political Dynamics of Flood Risk, Recovery and Response: A Report on the Findings of the Winter Floods Project. Exeter, UK: The University of Exeter, 2016:1-48. http://geography.exeter.ac.uk/media/universityofexeter/schoolofgeography/winterfloods/documents/Butler_et_al_2016_Socia_Political_DynamicsFloods.pdf (Accessed July 2018).

33. Twigger-Ross C, Kashefi E, Weldon S, et al. Flood Resilience Community Pathfinder Evaluation - Rapid Evidence Assessment. London, UK: Department for Environment, Food and Rural Affairs, 2014. https://nationalfloodforum.org.uk/wp-content/uploads/2017/04/Flood-Resilience-Community-Pathfinder-Evaluation_Rapid-Evidence-Assessment.pdf (Accessed September 2018).

34. Devlin NJ, Brooks R. EQ-5D and the EuroQol Group: Past, Present and Future. *Appl Health Econ Health Policy* 2017;15(2):127-37.

35. Munro A, Kovats RS, Rubin GJ, et al. Effect of evacuation and displacement on the association between flooding and mental health outcomes: a cross-sectional analysis of UK survey data. *Lancet Planet Health* 2017;1(4):e134-e41.

For peer review only

For peer review only - http://bmjopen.bmj.com/site/about/guidelines.xhtml

Appendix I. Socio-demographic characteristics of participants according to exposure to flooding

Characteristic	All participants (n=531)*	Exposure group		
		Unaffected (n=90)	Disrupted (n=322)	Flooded (n=119)
**Median age (IQR)**	62.4 (49.1 to 72.1)	64.0 (53.9 to 76.8)	61.9 (46.7 to 71.6)	61.3 (48.3 to 71.2)
**Sex**				
Male	217 (41.2%)	55 (61.1%)	113 (35.5%)	49 (41.2%)
Female	310 (58.8%)	35 (38.9%)	205 (64.5%)	70 (58.8%)
**Ethnicity**				
White	523 (99.6%)	88 (100%)	316 (99.4%)	119 (100%)
Other	2 (0.4%)	0 (0%)	2 (0.6%)	0 (0%)
**Marital Status**				
Married/civil partners/cohabiting	340 (64.6%)	68 (76.4%)	196 (61.6%)	76 (63.9%)
Separated/divorced/widowed	127 (24.1%)	16 (18.0%)	80 (25.2%)	31 (26.1%)
Single/other	59 (11.2%)	5 (5.6%)	42 (13.2%)	12 (10.1%)
**Housing tenure**				
Owner/joint owner/family owned	433 (82.3%)	73 (82.0%)	262 (82.4%)	98 (82.4%)
Other	93 (17.7%)	16 (18.0%)	56 (17.6%)	21 (17.6%)
**Employment**				
Employed	252 (47.8%)	36 (40.4%)	155 (48.4%)	61 (51.7%)
Retired	238 (45.2%)	47 (52.8%)	144 (45.0%)	47 (39.8%)
Other	37 (7.0%)	6 (6.7%)	21 (6.6%)	10 (8.5%)
**Education level**				
Degree or above	235 (44.8%)	29 (32.6%)	153 (%)	53 (44.9%)
Below degree level	172 (32.8%)	32 (36.0%)	102 (32.2%)	38 (32.2%)
Other	46 (8.8%)	8 (9.0%)	29 (9.2%)	9 (7.6%)
None (no formal qualifications)	71 (13.5%)	20 (22.5%)	33 (10.4%)	18 (15.3%)
**English deprivation quintile**				
1 (least deprived)	49 (9.4%)	17 (19.1%)	30 (9.4%)	2 (1.7%)
2	221 (42.2%)	38 (42.7%)	138 (43.4%)	45 (38.5%)
3	202 (38.6%)	25 (28.1%)	114 (35.9%)	63 (53.9%)
4 and 5**	52 (9.9%)	9 (10.1%)	36 (11.3%)	7(6.0%)
**Long-term illness**				
Yes	394 (76.5%)	69 (80.2%)	241 (77.0%)	84 (72.4%)
No	121 (23.5%)	17(19.8%)	72 (23.0%)	32 (27.6%)

Note: proportions calculated among those with known information on the characteristic of interest
*With known exposure status
**The two most deprived quintiles were combined due to small numbers

For peer review only

Appendix II. EQ-5D-5L health-related dimensions according to exposure to flooding

EQ-5D-5L dimension	Level	Exposure to flooding					
		All	Unaffected	Disrupted	Flooded (all)	Single flooded	Repeat flooded
		n (%)	n (%)	n (%)	n (%)	n (%)	n (%)
Mobility problems	No	391 (75.2%)	65 (73.9%)	240 (76.4%)	86 (74.6%)	41 (75.9%)	45 (70.3%)
	Slight	59 (11.3%)	11 (12.5%)	36 (11.5%)	11 (9.3%)	1 (1.9%)	10 (15.6%)
	Moderate	49 (9.4%)	8 (9.1%)	25 (8.0%)	16 (13.6%)	10 (18.5%)	6 (9.4%)
	Severe/unable*	22 (4.2%)	4 (4.5%)	13 (4.1%)	5 (4.2%)	2 (3.7%)	3 (4.7%)
Self-care problems	No	482 (92.7%)	84 (95.5%)	290 (93.0%)	108 (91.5%)	49 (90.7%)	59 (92.2%)
	Slight	18 (3.5%)	2 (2.3%)	13 (4.1%)	3 (2.5%)	1 (1.9%)	2 (3.1%)
	Moderate	16 (3.1%)	2 (2.3%)	8 (2.5%)	6 (5.1%)	4 (7.4%)	2 (3.1%)
	Severe/unable*	4 (0.8%)	0 (0%)	3 (1.0%)	1 (0.8%)	0 (0%)	1 (1.6%)
Activity problems	No	403 (77.5%)	71 (80.7%)	247 (78.7%)	85 (72.0%)	37 (68.5%)	48 (75.0%)
	Slight	55 (10.6%)	9 (10.2%)	32 (10.2%)	14 (11.9%)	7 (13.0%)	7 (10.9%)
	Moderate	48 (9.2%)	7 (8.0%)	28 (8.9%)	13 (11.0%)	8 (14.8%)	5 (7.8%)
	Severe/unable*	14 (2.7%)	1 (1.1%)	7 (2.2%)	6 (5.1%)	2 (3.7%)	4 (6.3%)
Pain/discomfort	No	311 (59.8%)	51 (58.0%)	194 (61.8%)	66 (55.9%)	29 (53.7%)	37 (57.8%)
	Slight	122 (23.5%)	25 (28.4%)	67 (21.3%)	30 (25.4%)	15 (28.8%)	15 (23.4%)
	Moderate	63 (12.1%)	8 (9.1%)	39 (12.4%)	16 (13.6%)	7 (13.0%)	9 (14.1%)
	Severe/extreme*	24 (4.6%)	4 (4.5%)	14 (4.5%)	6 (5.1%)	3 (5.6%)	3 (4.7%)
Anxiety/depression	No	347 (66.7%)	71 (80.7%)	232 (73.9%)	44 (37.3%)	19 (35.2%)	25 (39.1%)
	Slight	96 (18.5%)	13 (14.8%)	53 (16.9%)	30 (25.4%)	18 (33.3%)	12 (18.8%)
	Moderate	60 (11.5%)	3 (3.4%)	23 (7.3%)	34 (28.8%)	14 (25.9%)	20 (31.3%)
	Severe/extreme*	17 (3.3%)	1 (1.1%)	6 (1.9%)	10 (8.5%)	3 (5.6%)	7 (10.9%)

*Groups combined due to small numbers

For peer review only

STROBE Statement—Checklist of items that should be included in reports of ***cross-sectional studies***

	Item No	Recommendation
**Title and abstract**	1	(*a*) Indicate the study's design with a commonly used term in the title or the abstract – p.2
		(*b*) Provide in the abstract an informative and balanced summary of what was done and what was found – p.2
**Introduction**		
Background/rationale	2	Explain the scientific background and rationale for the investigation being reported - p.4
Objectives	3	State specific objectives, including any prespecified hypotheses – p.4
**Methods**		
Study design	4	Present key elements of study design early in the paper – p.5-6
Setting	5	Describe the setting, locations, and relevant dates, including periods of recruitment, exposure, follow-up, and data collection – p.5-6
Participants	6	(*a*) Give the eligibility criteria, and the sources and methods of selection of participants – p.5
Variables	7	Clearly define all outcomes, exposures, predictors, potential confounders, and effect modifiers. Give diagnostic criteria, if applicable – p.5-6
Data sources/ measurement	8*	For each variable of interest, give sources of data and details of methods of assessment (measurement). Describe comparability of assessment methods if there is more than one group – p.5-6
Bias	9	Describe any efforts to address potential sources of bias – p.5
Study size	10	Explain how the study size was arrived at – p.5
Quantitative variables	11	Explain how quantitative variables were handled in the analyses. If applicable, describe which groupings were chosen and why – p.5-6
Statistical methods	12	(*a*) Describe all statistical methods, including those used to control for confounding – p.6
		(*b*) Describe any methods used to examine subgroups and interactions – p.6
		(*c*) Explain how missing data were addressed – p.6
		(*d*) If applicable, describe analytical methods taking account of sampling strategy N/A
		(*e*) Describe any sensitivity analyses – N/A
**Results**		
Participants	13*	(a) Report numbers of individuals at each stage of study—eg numbers potentially eligible, examined for eligibility, confirmed eligible, included in the study, completing follow-up, and analysed – p.6-7
		(b) Give reasons for non-participation at each stage – N/A
		(c) Consider use of a flow diagram – not necessary
Descriptive data	14*	(a) Give characteristics of study participants (eg demographic, clinical, social) and information on exposures and potential confounders – p.6-7 and Appendix I
		(b) Indicate number of participants with missing data for each variable of interest – p.6-7
Outcome data	15*	Report numbers of outcome events or summary measures – p.6-7
Main results	16	(*a*) Give unadjusted estimates and, if applicable, confounder-adjusted estimates and their precision (eg, 95% confidence interval). Make clear which confounders were adjusted for and why they were included – p.7 and Tables 1, 2, 3 and 4

For peer review only - http://bmjopen.bmj.com/site/about/guidelines.xhtml
For peer review only

		(*b*) Report category boundaries when continuous variables were categorized – p.5
		(*c*) If relevant, consider translating estimates of relative risk into absolute risk for a meaningful time period – N/A
Other analyses	17	Report other analyses done—eg analyses of subgroups and interactions, and sensitivity analyses – N/A
**Discussion**		
Key results	18	Summarise key results with reference to study objectives – p.8
Limitations	19	Discuss limitations of the study, taking into account sources of potential bias or imprecision. Discuss both direction and magnitude of any potential bias – p.8-9
Interpretation	20	Give a cautious overall interpretation of results considering objectives, limitations, multiplicity of analyses, results from similar studies, and other relevant evidence – p8-9
Generalisability	21	Discuss the generalisability (external validity) of the study results – p.8-9
**Other information**		
Funding	22	Give the source of funding and the role of the funders for the present study and, if applicable, for the original study on which the present article is based – p.11

*Give information separately for exposed and unexposed groups.

Note: An Explanation and Elaboration article discusses each checklist item and gives methodological background and published examples of transparent reporting. The STROBE checklist is best used in conjunction with this article (freely available on the Web sites of PLoS Medicine at http://www.plosmedicine.org/, Annals of Internal Medicine at http://www.annals.org/, and Epidemiology at http://www.epidem.com/). Information on the STROBE Initiative is available at www.strobe-statement.org.

For peer review only

For peer review only - http://bmjopen.bmj.com/site/about/guidelines.xhtml

BMJ Open

Impact of repeat flooding on mental health and health-related quality of life: a cross-sectional analysis of the English National Study of Flooding and Health

Journal:	*BMJ Open*
Manuscript ID	bmjopen-2019-031562.R1
Article Type:	Original research
Date Submitted by the Author:	06-Aug-2019
Complete List of Authors:	French, Clare; University of Bristol, Population Health Sciences, Bristol Medical School<br>Waite, Thomas; Public Health England, National Infection Service<br>Armstrong, Ben; London School of Hygiene and Tropical Medicine<br>Rubin, GJ; King's College London, NIHR Health Protection Research Unit in Emergency Preparedness and Response, Department of Psychological Medicine, Weston Education Centre<br>National Study of Flooding and Health Study Group, English ; Public Health England<br>Beck, Charles; Public Health England, Field Service South West, National Infection Service; Bristol Medical School, NIHR Health Protection Unit in Evaluation of Interventions, Population Health Sciences<br>Oliver, Isabel; Public Health England, Field Service South West, National Infection Service; Bristol Medical School, NIHR Health Protection Unit in Evaluation of Interventions, Population Health Sciences
**Primary Subject Heading**:	Epidemiology
Secondary Subject Heading:	Mental health, Public health
Keywords:	EPIDEMIOLOGY, PUBLIC HEALTH, MENTAL HEALTH, Health-related quality of life, Flooding

SCHOLARONE™
Manuscripts

Impact of repeat flooding on mental health and health-related quality of life: a cross-sectional analysis of the English National Study of Flooding and Health

Clare E French[1], Thomas D Waite[2], Ben Armstrong[3], G. James Rubin[4], English National Study of Flooding and Health Study Group, Charles R Beck[1,5] Isabel Oliver[1,5]

[1] NIHR Health Protection Unit in Evaluation of Interventions, Population Health Sciences, Bristol Medical School, University of Bristol, BS8 2PS, UK

[2] National Infection Service, Public Health England, London, UK

[3] NIHR Health Protection Research Unit in Environmental Change and Health at the London School of Hygiene and Tropical Medicine, London, UK

[4] Department of Psychological Medicine, Weston Education Centre, King's College London NIHR Health Protection Research Unit in Emergency Preparedness and Response, London, UK

[5] Field Service South West, National Infection Service, Public Health England, Bristol, UK

Corresponding author: Clare French, Population Health Sciences, Bristol Medical School, Canynge Hall, 39 Whatley Road, Bristol BS8 2PS, UK. Email: clare.french@bristol.ac.uk. Tel: +44(0)117 3313925

Word count: 3,262

For peer review only - http://bmjopen.bmj.com/site/about/guidelines.xhtml

ABSTRACT

Objective To assess the association between flooding/repeat flooding and: i) psychological morbidity (anxiety, depression, post-traumatic stress disorder [PTSD]) and ii) health-related quality of life (HRQoL) at six months post-flooding.

Design Cross-sectional analysis of data from the English National Study of Flooding and Health.

Setting Cumbria, England.

Participants Questionnaires were sent to 2500 residential addresses at six months post-flooding; 590 people responded.

Outcomes Probable depression was assessed using the Patient Health Questionnaire (PHQ-2); probable anxiety using the Generalised Anxiety Disorder scale (GAD-2); and probable PTSD using the short-form PTSD checklist (PCL-6). Health-related quality of life (HRQoL) was assessed using the EQ-5D-5L.

Mental health outcomes were analysed using logistic regression; HRQoL dimensions using ordinal regression; and summary index/VAS scores using linear regression.

Results One hundred and nineteen participants had been flooded, over half of whom were experiencing a repeat flooding event (54%; n=64).

Mental health outcomes were elevated among flooded compared with unaffected participants (adjusted odds ratio for probable depression: 7.77, 95%CI: 1.51 to 40.13; anxiety: 4.16, 95%CI: 1.18 to 14.70; PTSD: 14.41, 95%CI: 3.91 to 53.13). The prevalence of depression was higher among repeat compared with single flooded participants, but this was not significant after adjustment. There was no difference in levels of anxiety or PTSD.

Compared with unaffected participants, those flooded had lower EQ-5D-5L index scores (adjusted coefficient: -0.06, 95% CI: -0.12 to -0.01) and lower self-rated health scores (adjusted coefficient: -6.99, 95% CI: -11.96 to -2.02). There was, however, little difference in HRQoL overall between repeat and single flooded participants.

Conclusions Interventions are needed to help minimise the impact of flooding on people's mental health and HRQoL.

For peer review only

For peer review only - http://bmjopen.bmj.com/site/about/guidelines.xhtml

Strengths and limitations of this study

- There is currently very little research exploring the impact of repeat flooding on mental health or health-related quality of life – our research addresses a gap in the evidence
- We use data from the English National Study of Flooding and Health – a large, robust study with proven methodology
- Three key mental health outcomes (depression, anxiety, post-traumatic stress disorder) as well as health-related quality of life were assessed, all using validated instruments
- The tools used to assess psychological outcomes are indictive of probable diagnoses rather than being clinically diagnostic
- Information was not available on the number or timing previous flooding events

For peer review only

For peer review only - http://bmjopen.bmj.com/site/about/guidelines.xhtml

INTRODUCTION

Flooding can have a range of effects on people's health, both physical and psychological.[1-5] Given projections that both the frequency and severity of flooding events will continue to increase in the future,[6-9] populations living in flood-susceptible areas may be exposed to multiple flooding events. It is important that the impact of flooding, and repeat flooding, on people's health and wellbeing is thoroughly understood. This will help inform public health actions to mitigate adverse health consequences of future flooding events.

Beyond the acute health consequences of flooding, such as injuries and infections, the longer-term health consequences are thought to be primarily psychological.[6] The English National Study of Flooding and Health began in 2015 to assess the impact of widespread flooding in southern England. The study has assessed the impact of flooding and related disruption on mental health and wellbeing over several years. Data obtained one year after flooding showed higher levels of depression, anxiety and post-traumatic stress disorder (PTSD) in participants whose homes had been flooded, and to a lesser extent, those whose lives had been disrupted by flooding compared to those who were unaffected.[10] Follow-up of participants at two years post-flooding demonstrated that mental health consequences had waned but were still noticeably higher in affected groups.[11] The impact of flooding on health-related quality of life (HRQoL) has not yet been reported on.

Several factors are known to be associated with the likelihood of experiencing adverse mental health outcomes post-flooding. These include socio-demographic characteristics such as age, gender, socio-economic and education statuses, and factors related to the nature of flooding e.g. depth and duration.[12] Secondary stressors, including problems with relationships and loss of sentimental items have been associated with poor psychological outcomes.[13] Meanwhile, societal-level factors such as social cohesion and support, as well as individual-level factors such as positive/proactive coping strategies can be protective against the development of mental ill health in the aftermath of flooding.[2,14] Although there is less published research on the association between flooding and wellbeing/quality of life, the available evidence indicates that effects similarly occur through a complex set of inter-related pathways.[15]

The UK Climate Change Risk Assessment 2017 highlighted flooding as a risk to people, communities and buildings that requires more action.[16] More research is required to assess and understand how these risks are best tackled. There is currently very limited research exploring the impact of experiencing repeated flooding events on mental health and/or HRQoL, and the evidence to-date is inconclusive.[14,17-19]

In early December 2015, exceptionally heavy and persistent rainfall associated with storm "Desmond", resulted in widespread flooding in Cumbria and other parts of northern Britain. Thousands of homes and businesses across Cumbria were flooded, and tens of thousands left without power.[20,21] Cumbria is an area that has been repeatedly affected by flooding - the region had experienced severe flooding just six years previously in 2009, and also in 2005.[21]

The objective of this study is to assess the association between flooding/repeat flooding and: i) psychological morbidity (probable anxiety, probable depression, probable PTSD) and ii) HRQoL, in the Cumbria region at six months post-flooding.

For peer review only - http://bmjopen.bmj.com/site/about/guidelines.xhtml

METHODS

Study conduct

Details on the design and conduct of the English National Study of Flooding and Health have been published previously.[10] Recruitment and data collection in Cumbria largely mirrored the original survey conducted elsewhere. Recruitment packs (including consent forms, study information sheets and questionnaires) were sent to 2500 residential households in postcode areas known to have been affected by the 2015/16 floods within four local authority areas (Allerdale, Carlisle, Eden, South Lakeland). The sample size was determined with the aim of sampling approximately 50% of flooded households. All adults in each household were invited to participate. Packs were sent out approximately six months post-flooding (May 2016), with two rounds of reminder letters sent to non-responders. Invitations were sent by post and participants had the option to respond either by post or online. Questionnaires were similar to those used for the original survey, with some minor clarification to the wording of questions, and the addition of the EQ-5D-5L instrument to assess HRQoL.

Participants were asked about their exposure to the 2015/16 floods, and whether they had been previously flooded. They were categorised as:

- Unaffected - those who experienced no flooding or disruption during the 2015/16 floods
- Disrupted - those who did not have floodwater in liveable areas of their homes but reported experiencing disruption due to the floods (e.g. floodwater in non-liveable areas such as a garage, disruption to loss of utilities, loss of access to services)
- Flooded - those who reported having floodwater in one or more liveable rooms of their home (e.g. living room, kitchen, bedroom). For analyses of repeat flooding this group was further sub-categorised into:
    - Repeat flooded - those who reported having been previously flooded (either in the same or a previous home),
    - Single flooded - those who reported not having been previously flooded.

Demographic information collected included date of birth, sex, ethnicity, long-term illness or disability, education, employment and marital statuses. Postcodes of residence were used to assign Lower Super Output Area deprivation scores based on the Index of Multiple Deprivation.[22] These were ranked according to scores for all of England and categorised into quintiles.

Mental health outcomes were assessed using validated instruments - probable depression using the Patient Health Questionnaire (PHQ-2) depression sub-scale; probable anxiety using the Generalised Anxiety Disorder scale (GAD-2); and probable PTSD using the short-form PTSD checklist (PCL-6). Cut-off scores, used to indicate the likely presence of the condition, were ≥3 for PHQ-2/GAD-2 and ≥14 for PCL-6.[23-25] We also generated a combined variable indicating the presence of 'any' of these probable conditions.

HRQoL was assessed using the EQ-5D-5L. This is a validated two-part instrument. The first part is a descriptive system assessing five health-related dimensions (mobility, self-care, usual activities, pain/discomfort and anxiety/depression) on a five-point scale (no problems, slight problems, moderate problems, severe problems and extreme problems). Information on these five health states was converted into a single index value. The second part is a visual analogue scale (VAS) which records the patient's self-rated health on a vertical scale from marked from 0

For peer review only - http://bmjopen.bmj.com/site/about/guidelines.xhtml

to 100 where the endpoints are 'The best health you can imagine' and 'The worst health you can imagine'.[26]

SelectSurvey (ClassApps, USA) was used to develop the online questionnaire and capture online responses. Data returned on paper forms were double-entered using EpiData (EpiData Association, Denmark) and cross-checked to identify any errors.

Data analysis

Data were analysed in STATA 13.1 (StataCorp, USA). Participants who did not provide enough information to enable an exposure status to be assigned to them were excluded from analyses by exposure group. For analyses of mental health outcomes, participants who did not complete an outcome instrument were excluded from analyses of that outcome only. HRQoL analyses were restricted to those with complete information on both parts of the tool.

Mental health outcomes were analysed using binary logistic regression. For analyses of HRQoL, the EQ-5D-5L descriptive system ordered categorical variables were analysed using ordinal logistic regression. The proportional odds assumption was checked for each outcome using approximate likelihood-ratio tests. Continuous variables (EQ-5D-5L index score and VAS) both had left-skewed distributions and are thus presented as medians and inter-quartile ranges (IQRs). Medians were compared using the Mann Whitney U test and adjusted analyses were conducted using linear regression with robust standard errors to allow for the skewed distribution of the dependent variable.

Potential socio-demographic confounders of the association between flooding and mental health outcomes have been previously identified (age group, sex, local authority, ethnicity, marital status, education level, employment, deprivation score and pre-existing illness).[10] All (except ethnicity, due to very small numbers in the non-white ethnic group) were adjusted for *a priori* in all multivariable analyses for consistency and comparability between models.

Patient and public involvement

A community flooding action group was involved in the design of the study. Volunteers identified by the National Flood Forum piloted early versions of the questionnaires, and the National Flood Forum Chief Executive is a member of our stakeholder group. Participants were invited to join a study group to support the project and keep track of its progress. Study findings are being disseminated to participants via reports published online.

RESULTS

Response rates

Completed questionnaires were received from 590 participants (household response rate: 24.4%). More than one individual responded from 19 households giving a total of 628 participating individuals. Most participants responded using the paper questionnaire, with 7.0% (n=44) responding online. Subsequent analyses were conducted on the 531 participants who provided sufficient information to enable an exposure status to be assigned to them.

Participant characteristics

The socio-demographic characteristics of study participants are provided in Appendix I. Overall, the median age was 62 years (inter-quartile range [IQR]: 49 to 72 years), 58.8% were female,

For peer review only - http://bmjopen.bmj.com/site/about/guidelines.xhtml

99.6% were of white ethnicity, 51.6% lived in areas belonging to the two least deprived quintiles and 76.5% had a long-term illness or disability.

Exposure to flooding and psychological morbidity

Overall, 16.9% of participants were unaffected by the floods, 60.6% were disrupted and 22.4% were flooded.

After adjusting for socio-demographic factors, there was an elevated odds of each mental health outcome among flooded participants compared to those unaffected (adjusted odds ratio [aOR] for probable depression: 7.77, 95% confidence interval [CI]: 1.51 to 40.13; anxiety: 4.16, 95% CI: 1.18 to 14.70; PTSD: 14.41, 95% CI: 3.91 to 53.13; and for 'any' of these mental health outcomes: 11.81, 95% CI: 3.99-34.96). The adjusted odds of probable depression, PTSD and any outcome (but not anxiety) were also somewhat raised for those who experienced disruption due to flooding but not significantly so (Table 1).

Of those flooded during the 2015/16 floods, over half (54%; 64/119) had been previously flooded (60 at the same home and four at a previous home). The prevalence of probable depression was elevated among those exposed to repeated, as compared with a single flooding event, but the difference reduced on adjustment for potential confounders, and was not significant. For probable anxiety and PTSD there was little difference in prevalence among people who experienced single and repeat flooding, either before or after adjustment (Table 1).

Exposure to flooding and HRQoL

Most participants (97.9%, n=520) had complete information on both parts of the EQ-5D-5L.

Index scores

Median EQ-5D-5L index scores are presented in Table 2. In adjusted analyses flooded participants (all) had lower EQ-5D-5L index scores than unaffected participants (adjusted coefficient: -0.06, 95% CI: -0.12 to -0.01). There was little evidence that scores differed between repeat and single flooded participants.

Health-related dimensions

The level of problems reported by participants on each dimension, according to exposure to flooding are provided in Appendix II.

Flooded participants were more likely to have higher anxiety/depression scores than those unaffected (aOR: 8.59, 95% CI:4.02 to 18.36; Table 3), also more self-care and activity problems, though not significantly so. There was little evidence that the flooded group had more mobility problems or pain/discomfort than those unaffected. There was little evidence of a difference between repeat and single flooded participants on any dimension (Table 3). There was no evidence against the proportional odds assumption for any of the five dimensions.

VAS scores

Median VAS scores are presented in Table 4. Scores were 78 (IQR 69 to 90) for flooded participants, notably lower than for those unaffected (90, IQR: 75 to 93). This difference remained after adjustment (adjusted coefficient of VAS scores among flooded vs. unaffected participants: -6.99, 95% CI: -11.96 to -2.02). There was little evidence of a difference in VAS scores between the repeat and single flooded groups. For analyses of both index scores and

For peer review only - http://bmjopen.bmj.com/site/about/guidelines.xhtml

VAS scores the key assumptions of linear regression were met; robust standard errors were used which dealt with the non-normality of the data.

DISCUSSION

This study provides strong evidence of an association between flooding, be it a single or repeat episode, and probable mental health outcomes. Flooded participants also tended to have poorer HRQoL than those unaffected by the floods. Analyses of the dimension-specific descriptive EQ-5D-5L system indicate that anxiety/depression may be an important driver of this association. There was little evidence of a difference in either probable mental health outcomes or HRQoL between repeat and single flooded participants, although data were suggestive of a greater level of depression in those repeatedly flooded.

The UK Climate Change Risk Assessment 2017 identifies flooding as a major risk to people and the built environment; more action is needed to mitigate this risk.[16] The UK government has committed to actions, such as ensuring that people have good access to information to assess the risks, including health risks, of flooding; helping people to make their properties more resistant to flooding; and ensuring plans are in place to predict and respond to flooding incidents.[27] Our research should be used to inform that government action - although we focus on the impact of flooding on health and wellbeing, the evidence presented here supports the case for good town planning, flood and coastal risk management, and the implementation of the 25-year Environment Plan.[27]

The overall associations between being flooded (vs. unaffected) and mental health outcomes were consistent with those measured in the cohort affected by the floods in the winter of 2013/14 in Southern England.[10] Those floods largely affected affluent areas potentially limiting the generalisability of the findings beyond that population. Participants from Cumbria resided in areas of comparatively greater deprivation; our findings thus demonstrate that the increased risks of mental health outcomes following flooding also pertain to those living in more deprived areas. This is consistent with low income having been previously reported as a known driver of vulnerability to the effects of flooding.[28] Of note, a strong association between flooding and anxiety/depression was found using the EQ-5D-5L, corroborating findings obtained using mental health outcome assessment tools.

There is limited previous research on the impact of flooding on HRQoL with which to draw comparisons, and we are not aware of any previously published comparable data on the impact of repeat flooding specifically. A mixed-methods study in two UK regions found that flooding had a marked impact on wellbeing. This occurred via a complex and diverse range of interacting factors acting at both the individual and societal level.[15] Data from China showed poorer quality of life among those affected by floods compared with those unaffected, but the EQ-5D-5L tool was not used and the findings are not directly comparable.[29]

That over half of flooded participants in our sample had experienced repeat flooding is striking. It may be hypothesized that this group will suffer greater mental health and HRQoL effects than those experiencing single event due to the cumulative effects of these experiences. However, our research indicates that any association may be more nuanced. The data were suggestive of higher levels of depression, but not other mental health outcomes or overall HRQoL, among repeat compared with single flooded participants. Of note, the number of events were small in some groups resulting in imprecise estimates – the data should thus be interpreted with some caution. The lack of association found our study does, however, concur with a recently published

For peer review only - http://bmjopen.bmj.com/site/about/guidelines.xhtml

Australian study which reported a general lack of association between exposure to repeat natural disasters and the broader psychiatric disorder spectrum.[30]

Repeat flooding may impact on mental health in a variety of ways, though the evidence is inconclusive.[14,17,18] At the individual level, experiencing a repeat episode of flooding may trigger memories of previous flooding episodes, intensifying the distress experienced.[17] Those who have been previously flooded may also experience anticipatory anxiety and stress about the prospect of future flooding.[31,32] Conversely, people's individual and collective coping capacity may be increased, and they may display greater resilience to future flooding events.[33] There is also evidence to suggest that previous exposure to disasters may have an 'inoculation' effects, particularly among older adults (the median age of our study population was 62 years).[19, 34-35] Meanwhile, factors acting at the community level may mitigate the risks of adverse consequences of future floods through the development or strengthening of community networks and increased social cohesion.[15,31,36] Finally, past flood events may have resulted in improvements to infrastructure, flood defences and response systems, reducing the extent of subsequent floods and the impact on people's lives.

General limitations of the National Study of Flooding and Health have been outlined elsewhere.[10] Our questionnaire response rate of 24% is in line with other similar postal surveys.[37,38] Although this relatively low response rate is a potential limitation of our study, it would only bias the observed associations between flooding and mental health / HRQoL if response was differential with respect to both flooding and health outcomes. Although this is a possibility, we don't have evidence to suggest that any such differential response would be substantial. Another limitation of these analyses specifically is that we do not have any information on the number, timing or extent of previous flooding experiences. Such information would be helpful in interpreting our findings and should be considered when designing future research studies on the health impacts of flooding.

Though validated instruments, the tools used to assess psychological outcomes are not clinically diagnostic. Our data therefore indicate 'probable' diagnoses which are likely an overestimate of diagnosable mental health conditions. Meanwhile, the EQ-5D-5L is a validated and widely used tool that has been previously used in UK populations.[39] The tool was well completed (98% of participants had complete data) indicating its acceptability in our study. However, some of the dimensions assessed may not be directly relevant to the types of illnesses that may occur as a consequence of flooding. At the same time, due to space constraints on the questionnaire, we were unable to assess all psychological variables that are of theoretical interest following repeat flooding. For example, anger might be elevated in flooded, and especially repeat flooded, participants and may be worthy of future investigation.

Our findings may not be generalisable to all populations affected by flooding (e.g. there were few participants of non-white ethnicity). However, all comparison groups were drawn from the same population and key socio-demographic confounding factors were adjusted for in the analyses.

Conclusions and recommendations

Flooding can have a profound effect on people's psychological health, and aspects of their HRQoL. Though we found little evidence that people exposed to repeat flooding were at increased risk of adverse outcomes compared with those exposed to a single event, it should be remembered that these individuals are at risk of experiencing adverse outcomes each time they experience flooding.

For peer review only - http://bmjopen.bmj.com/site/about/guidelines.xhtml
For peer review only

That over half of participants in this study were experiencing a repeat flooding event highlights the need for strengthening flood defences and mitigating risks of future flooding in areas at risk or previously affected. The UK's National Adaptation Programme for climate change recommends actions for reducing flood risk and harm from flooding including effective land use planning;[27] the evaluation of these planned actions should include an assessment of their impact on people's mental health and wellbeing.

Interventions are needed to help minimise the impact of flooding on mental health and wellbeing. These may include early warning systems which can reduce the effects of flooding on mental health,[40] and community-based therapeutic actions such as psychological first aid in the immediate aftermath of flood events. Further research on the effectiveness of these and other interventions to reduce the impact of flooding on people's health would be informative in shaping future intervention strategies.

Providing people with other approaches to reduce their susceptibility to the effects of flooding, such as efforts to increase community cohesion and develop support networks in preparedness for potential future flooding events, may also be beneficial. Finally, ensuring that adequate and effective mental health services are available for community members following flooding is vital.

For peer review only

For peer review only - http://bmjopen.bmj.com/site/about/guidelines.xhtml

Acknowledgments
We would like to thank the participants in this study and are grateful for the support we received from Colin Cox, Director of Public Health Cumbria County Council and the North West Public Health England Centre. We acknowledge the role of other members of the English National Study of Flooding and Health Study Group in the design of the National Study of Flooding and Health: Katerina Chaintarli, Angie Bone, Richard Amlôt, Sari Kovats, Mark Reacher and Giovanni Leonardi. We would also like to thank all PHE staff who contributed to study administration, set up and data entry.

Part of this work was presented at the Public Heath England Public Health Research and Science Conference 2018, Warwick, UK.

Ethics approval
Ethical approval for the study was granted by the Psychiatry, Nursing and Midwifery Research Ethics Subcommittee at King's College London [Reference PNM 1314 152]. All participants consented to participate in the study.

Data sharing
The datasets analysed in this study are available from Public Health England Field Epidemiology Service on reasonable request.

Competing interests
The authors declare that they have no competing interests.

Funding
The research was funded in part by the National Institute for Health Research Health Protection Research Units (NIHR HPRU) in Emergency Preparedness and Response at King's College London, Environmental Change at the London School of Hygiene and Tropical Medicine and Evaluation of Interventions at the University of Bristol, in partnership with Public Health England (PHE). The views expressed are those of the authors and not those of the NHS, the NIHR, The Department of Health or Public Health England.

Author contributions
CEF led the design and conduct of these analyses, with expert statistical advice from BA, and drafted the manuscript. English National Study of Flooding and Health Study Group members including named authors IO, TDW, BA, CRB and GJR designed the National Study of Flooding and Health and acquired the data. IO, TDW, BA, CRB and GJR contributed to the design of the analyses and the interpretation the findings. All authors contributed to the writing of the manuscript and approved it for submission.

Exclusive license
The Submitting Author accepts and understands that any supply made under these terms is made by BMJ to the Submitting Author unless you are acting as an employee on behalf of your employer or a postgraduate student of an affiliated institution which is paying any applicable article publishing charge ("APC") for Open Access articles. Where the Submitting Author wishes to make the Work available on an Open Access basis (and intends to pay the relevant APC), the terms of reuse of such Open Access shall be governed by a Creative Commons licence – details of these licences and which Creative Commons licence will apply to this Work are set out in our licence referred to above.

For peer review only - http://bmjopen.bmj.com/site/about/guidelines.xhtml

Table 1. Prevalence and odds ratios (crude and adjusted) of mental health outcomes according to exposure to flooding

	n/N (%)*	OR (95% CI)	aOR** † (95% CI)
**MODEL 1 - Comparing flooded and disrupted with unaffected participants**			
**Probable depression**			
Unaffected	2/83 (2.4%)	Ref	Ref
Disrupted	15/292 (5.1%)	2.19 (0.49 to 9.80)	1.38 (0.27 to 6.96)
Flooded	25/106 (23.6%)	12.50 (2.87 to 54.52)	7.77 (1.51 to 40.13)
**Probable anxiety**			
Unaffected	5/83 (6.0%)	Ref	Ref
Disrupted	19/289 (6.6%)	1.10 (0.40 to 3.03)	0.91 (0.27 to 3.08)
Flooded	24/107 (22.4%)	4.51 (1.64 to 12.41)	4.16 (1.18 to 14.70)
**Probable PTSD**			
Unaffected	3/87 (3.4%)	Ref	Ref
Disrupted	30/300 (10.0%)	3.11 (0.93 to 10.45)	2.05 (0.57 to 7.40)
Flooded	49/112 (43.8%)	21.78 (6.49 to 73.08)	14.41 (3.91 to 53.13)
**'Any' probable mental health outcome**			
Unaffected	6/82 (7.3%)	Ref	Ref
Disrupted	41/285 (14.4%)	2.13 (0.87 to 5.21)	1.88 (0.67 to 5.32)
Flooded	52/107 (48.6%)	11.98 (4.08 to 29.86)	11.81 (3.99 to 34.96)
**MODEL 2 - Comparing repeat with single flooded participants**			
**Probable depression**			
Single flooded	8/49 (16.3%)	Ref	Ref
Repeat flooded	17/57 (29.8%)	2.18 (0.85 to 5.61)	1.55 (0.44 to 5.46)
**Probable anxiety**			
Single flooded	11/50 (22.0%)	Ref	Ref
Repeat flooded	13/57 (22.8%)	1.05 (0.42 to 2.61)	0.89 (0.26 to 2.97)
**Probable PTSD**			
Single flooded	21/51 (41.2%)	Ref	Ref
Repeat flooded	28/61 (45.9%)	1.21 (0.57 to 2.57)	0.73 (0.27 to 1.97)
**'Any' probable mental health outcome**			
Single flooded	22/48 (45.8%)	Ref	Ref
Repeat flooded	30/59 (50.8%)	1.22 (0.57 to 2.62)	1.03 (0.39 to 2.73)

OR – odds ratio, aOR – adjusted odds ratio, CI – confidence interval

*N is the total number of participants in each exposure group for whom a complete answer to this outcome instrument was provided, and % is the proportion of those who have the relevant (probable) condition

For peer review only

**Adjusted for age group, sex, local authority, marital status, education level, employment, deprivation score and pre-existing illness

†Number of observations included in the multivariable models were: probable depression n=446, probable anxiety n=444, probable PTSD n=464, 'any' probable mental health outcome n=440. Both models included the same participants but the reference group differs. Odds ratios are only presented for the comparison of interest

For peer review only

Table 2. Univariable and multivariable analyses of the association between exposure to flooding and EQ-5D-5L index values

	Exposure group	Median (IQR) index value	Crude coefficient (95% CI)*	Adjusted coefficient (95% CI)* **
MODEL 1	Unaffected	1.00 (0.77 to 1.00)	Ref	Ref
	Disrupted	1.00 (0.77 to 1.00)	-0.00 (-0.52 to 0.04)	-0.00 (-0.44 to 0.04)
	Flooded	0.85 (0.69 to 1.00)	-0.08 (-0.14 to -0.02)	-0.06 (-0.12 to -0.01)
MODEL 2	Single flooded	0.84 (0.68 to 0.88)	Ref	Ref
	Repeat flooded	0.85 (0.69 to 1.00)	0.00 (-0.08 to 0.08)	0.02 (-0.05 to 0.09)

*Confidence intervals estimated using robust standard errors

**Adjusted for age group, sex, local authority, marital status, education level, employment, deprivation score and pre-existing illness

For peer review only

For peer review only - http://bmjopen.bmj.com/site/about/guidelines.xhtml

Table 3. Univariable and multivariable analyses of EQ-5D-5L health-related dimensions according to exposure to flooding

	OR (95% CI)	aOR (95% CI)* **
**MODEL 1 - Comparing flooded and disrupted with unaffected participants**		
**Mobility problems**		
Unaffected	Ref	Ref
Disrupted	0.87 (0.51 to 1.49)	0.98 (0.48 to 2.02)
Flooded	1.09 (0.59 to 2.02)	1.04 (0.44 to 2.46)
**Self-care problems†**		
Unaffected	Ref	Ref
Disrupted	1.73 (0.58 to 5.12)	1.61 (0.41 to 6.31)
Flooded	1.98 (0.60 to 6.52)	1.62 (0.33 to 7.91)
**Activity problems**		
Unaffected	Ref	Ref
Disrupted	1.15 (0.64 to 2.07)	1.59 (0.70 to 3.60)
Flooded	1.68 (0.87 to 3.25)	1.85 (0.72 to 4.79)
**Pain/discomfort**		
Unaffected	Ref	Ref
Disrupted	0.92 (0.58 to 1.46)	0.95 (0.54 to 1.68)
Flooded	1.14 (0.67 to 1.95)	1.01 (0.50 to 2.02)
**Anxiety/depression**		
Unaffected	Ref	Ref
Disrupted	1.51 (0.84 to 2.70)	1.46 (0.73 to 2.91)
Flooded	7.72 (4.11 to 14.50)	8.59 (4.02 to 18.36)
**MODEL 2 - Comparing repeat with single flooded participants**		
**Mobility problems**		
Single flooded	Ref	Ref
Repeat flooded	1.16 (0.52 to 2.63)	0.85 (0.28 to 2.54)
**Self-care problems†**		
Single flooded	Ref	Ref
Repeat flooded	0.82 (0.23 to 3.01)	0.42 (0.08 to 2.29)
**Activity problems**		
Single flooded	Ref	Ref
Repeat flooded	0.74 (0.33 to 1.64)	0.71 (0.24 to 2.11)
**Pain/discomfort**		
Single flooded	Ref	Ref
Repeat flooded	0.88 (0.43 to 1.77)	0.52 (0.21 to 1.26)
**Anxiety/depression**		
Single flooded	Ref	Ref
Repeat flooded	1.20 (0.62 to 2.32)	1.13 (0.51 to 2.51)

For peer review only

For peer review only - http://bmjopen.bmj.com/site/about/guidelines.xhtml

*Adjusted for age group, sex, local authority, marital status, education level, employment, deprivation score and pre-existing illness

**Number of observations included in all multivariable models was 484. Both models 1 and 2 included the same participants, but the reference group differed. Odds ratios are only presented for the comparison of interest

†Due to very small numbers of participants with ‘severe/extreme problems’ on the self-care dimension, the ‘severe/extreme’ and ‘moderate’ categories were combined

For peer review only

For peer review only - http://bmjopen.bmj.com/site/about/guidelines.xhtml

Table 4. Univariable and multivariable analyses of the association between exposure to flooding and EQ-5D-5L VAS scores

	Exposure group	Median (IQR) VAS score	Crude coefficient (95% CI)*	Adjusted coefficient (95% CI)* **
MODEL 1	Unaffected	90 (75 to 93)	Ref	Ref
	Disrupted	85 (75 to 90)	-2.18 (-6.15 to 1.79)	-1.77 (-5.45 to 1.91)
	Flooded	78 (69 to 90)	-8.66 (-13.66 to -3.67)	-6.99 (-11.96 to -2.02)
MODEL 2	Single flooded	75 (70 to 85)	Ref	Ref
	Repeat flooded	80 (65 to 90)	-0.64 (-7.78 to 6.50)	0.76 (-7.06 to 8.60)

*Confidence intervals estimated using robust standard errors

**Adjusted for age group, sex, local authority, marital status, education level, employment, deprivation score and pre-existing illness

For peer review only

References

1. Ahern M, Kovats RS, Wilkinson P, et al. Global health impacts of floods: epidemiologic evidence. *Epidemiol Rev* 2005;27:36-46.

2. Fernandez A, Black J, Jones M, et al. Flooding and mental health: a systematic mapping review. *PLoS One* 2015;10(4):e0119929.

3. Saulnier DD, Brolin Ribacke K, von Schreeb J. No Calm After the Storm: A Systematic Review of Human Health Following Flood and Storm Disasters. *Prehosp Disaster Med* 2017;32(5):568-79.

4. Stanke C, Murray V, Amlot R, et al. The effects of flooding on mental health: Outcomes and recommendations from a review of the literature. *PLoS Currents* 2012; e4f9f1fa9c3cae. doi:10.1371/4f9f1fa9c3cae.

5. Zhong S, Yang L, Toloo S, et al. The long-term physical and psychological health impacts of flooding: A systematic mapping. *Sci Total Environ* 2018;626:165-94.

6. World Health Organization Regional Office for Europe. Floods in the WHO European Union: health effects and their prevention. Copenhagen, Denmark: World Health Organization, 2013:1-146. http://www.euro.who.int/__data/assets/pdf_file/0020/189020/e96853.pdf (Accessed September 2018).

7. European Environment Agency, European Commission Joint Research Centre and the World Health Organization. Impacts of Europe's changing climate - 2008 indicator-based assessment. Joint EEA-JRC-WHO report. Copenhagen, Denmark: European Environment Agency, European Commission Joint Research Centre and the World Health Organization, 2008:1-246. https://ec.europa.eu/jrc/sites/jrcsh/files/jrc_reference_report_2008_09_climate_change.pdf (Accessed September 2018).

8. Alfieri L, Bisselink B, Dottori F, et al. Global projections of river flood risk in a warmer world. *Earths Future* 2017;5(2):171-82.

9. Sayers PB, Horritt M, Penning-Rowsell E, et al. Climate Change Risk Assessment 2017: Projections of future flood risk in the UK. London, UK: Committee on Climate Change, 2017. https://www.theccc.org.uk/wp-content/uploads/2015/10/CCRA-Future-Flooding-Main-Report-Final-06Oct2015.pdf (Accessed October 2018).

10. Waite TD, Chaintarli K, Beck CR, et al. The English national cohort study of flooding and health: cross-sectional analysis of mental health outcomes at year one. *BMC Public Health* 2017;17(1):129.

11. Jermacane D, Waite TD, Beck CR, et al. The English National Cohort Study of Flooding and Health: the change in the prevalence of psychological morbidity at year two. *BMC Public Health* 2018;18(1):330.

12. Lowe D, Ebi KL, Forsberg B. Factors increasing vulnerability to health effects before, during and after floods. *Int J Environ Res Public Health* 2013;10(12):7015-67.

13. Tempest EL, English National Study on F, Health Study G, et al. Secondary stressors are associated with probable psychological morbidity after flooding: a cross-sectional analysis. *Eur J Public Health* 2017;27(6):1042-47.

14. Greene G, Paranjothy S, Palmer SR. Resilience and Vulnerability to the Psychological Harm From Flooding: The Role of Social Cohesion. *Am J Public Health* 2015;105(9):1792-5.

15. Walker-Springett K, Butler C, Adger WN. Wellbeing in the aftermath of floods. *Health Place* 2017;43:66-74.

For peer review only - http://bmjopen.bmj.com/site/about/guidelines.xhtml

16. Committee on Climate Change. UK Climate Change Risk Assessment 2017. Synthesis report: priorities for the next five years. London, UK: Committee on Climate Change, 2016. https://www.theccc.org.uk/wp-content/uploads/2016/07/UK-CCRA-2017-Synthesis-Report-Committee-on-Climate-Change.pdf (Accessed October 2018).

17. Lamond J. The role of flood memory in the impact of repeat flooding on mental health. In: Proverbs D. and Brebbia, C., eds. *Flood Recovery, Innovation and Response IV*. Ashurst, UK: WIT Press, 2014. https://www.witpress.com/books/978-1-84564-784-1 (Accessed August 2018)

18. Mason V, Andrews H, Upton D. The psychological impact of exposure to floods. *Psychol Health Med* 2010;15(1):61-73.

19. Norris FH, Murrell SA. Prior experience as a moderator of disaster impact on anxiety symptoms in older adults. *Am J Community Psychol* 1988;16(5):665-83.

20. Met Office. Flooding in Cumbria December 2015. Secondary Flooding in Cumbria December 2015. https://www.metoffice.gov.uk/climate/uk/interesting/december2015. (Accessed July 2018).

21. Miller JD, Kjeldsen TR, Hannaford J, et al. A hydrological assessment of the November 2009 floods in Cumbria, UK. *Hydrology Research* 2013;44(1):180-97.

22. Department for Communities and Local Government. English indices of deprivation 2015. Secondary English indices of deprivation 2015. https://www.gov.uk/government/statistics/english-indices-of-deprivation-2015 (Accessed May 2018).

23. Kroenke K, Spitzer RL, Williams JB. The Patient Health Questionnaire-2: validity of a two-item depression screener. *Med Care* 2003;41(11):1284-92.

24. Kroenke K, Spitzer RL, Williams JB, et al. Anxiety disorders in primary care: prevalence, impairment, comorbidity, and detection. *Ann Intern Med* 2007;146(5):317-25.

25. Lang A, Stein M. An abbreviated PTSD checklist for use as a screening instrument in primary care. *Behaviour Research and Therapy* 2005;43(5):585-94.

26. van Reenen M, Janssen B. EQ-5D-5L User Guide. Version 2.1,2015:1-28. https://euroqol.org/wp-content/uploads/2016/09/EQ-5D-5L_UserGuide_2015.pdf (Accessed June 2018)

27. Department for Environment, Food and Rural Affairs. The National Adaptation Programme and the Third Strategy for Climate Adaptation Reporting. London, UK: Department for Environment, 2018. https://assets.publishing.service.gov.uk/government/uploads/system/uploads/attachment_data/file/727252/national-adaptation-programme-2018.pdf (Accessed September 2018).

28. Sayers PB, Horritt M, Penning Rowsell E, et al. Present and future flood vulnerability, risk and disadvantage: A UK scale assessment. A report for the Joseph Rowntree Foundation. Watlington, UK: Sayers and Partners LLP, 2017:1-98. http://www.sayersandpartners.co.uk/uploads/6/2/0/9/6209349/sayers_2017__present_and_future_flood_vulnerability_risk_and_disadvantage_-_final_report_-_uploaded_05june2017_printed_-_high_quality.pdf (Accessed September 2018).

29. Tan HZ, Luo YJ, Wen SW, et al. The effect of a disastrous flood on the quality of life in Dongting lake area in China. *Asia Pac J Public Health* 2004;16(2):126-32.

30. Reifels L, Mills K, Duckers MLA, O'Donnell ML. Psychiatric epidemiology and disaster exposure in Australia. *Epidemiol Psychiatr Sci* 2019;28(3):310-20.

For peer review only - http://bmjopen.bmj.com/site/about/guidelines.xhtml

31. Tapsell SM, Penning-Rowsell EC, Tunstall SM, et al. Vulnerability to flooding: health and social dimensions. *Philos Trans A Math Phys Eng Sci* 2002;360(1796):1511-25.

32. Tunstall S, Tapsell S, Green C, et al. The health effects of flooding: Social research results from England and Wales. *J Water Health* 2006;4(3):365-80.

33. Bulter C, Walker-Springett K, Adger WN, et al. Social and Political Dynamics of Flood Risk, Recovery and Response: A Report on the Findings of the Winter Floods Project. Exeter, UK: The University of Exeter, 2016:1-48. http://geography.exeter.ac.uk/media/universityofexeter/schoolofgeography/winterfloods/documents/Butler_et_al_2016_Socia_Political_DynamicsFloods.pdf (Accessed July 2018).

34. Knight BG, Gatz M, Heller K, Bengtson VL. Age and emotional response to the Northridge earthquake: a longitudinal analysis. *Psychol Aging* 2000;15(4):627-34.

35. Shrira A, Palgi Y, Hamama-Raz Y, Goodwin R, Ben-Ezra M. Previous exposure to the World Trade Center terrorist attack and posttraumatic symptoms among older adults following Hurricane Sandy. *Psychiatry* 2014;77(4):374-85.

36. Twigger-Ross C, Kashefi E, Weldon S, et al. Flood Resilience Community Pathfinder Evaluation - Rapid Evidence Assessment. London, UK: Department for Environment, Food and Rural Affairs, 2014. https://nationalfloodforum.org.uk/wp-content/uploads/2017/04/Flood-Resilience-Community-Pathfinder-Evaluation_Rapid-Evidence-Assessment.pdf (Accessed September 2018).

37. Lamond JE, Joseph RD, Proverbs DG. An exploration of factors affecting the long term psychological impact and deterioration of mental health in flooded households. *Environ Res* 2015;140:325-34.

38. Paranjothy S, Gallacher J, Amlot R, Rubin GJ, Page L, Baxter T, et al. Psychosocial impact of the summer 2007 floods in England. *BMC Public Health* 2011;11:145.

39. Devlin NJ, Brooks R. EQ-5D and the EuroQol Group: Past, Present and Future. *Appl Health Econ Health Policy* 2017;15(2):127-37.

40. Munro A, Kovats RS, Rubin GJ, et al. Effect of evacuation and displacement on the association between flooding and mental health outcomes: a cross-sectional analysis of UK survey data. *Lancet Planet Health* 2017;1(4):e134-e41.

For peer review only

For peer review only - http://bmjopen.bmj.com/site/about/guidelines.xhtml

Appendix I. Socio-demographic characteristics of participants according to exposure to flooding

Characteristic	All participants (n=531)*	Exposure group		
		Unaffected (n=90)	Disrupted (n=322)	Flooded (n=119)
**Median age (IQR)**	62.4 (49.1 to 72.1)	64.0 (53.9 to 76.8)	61.9 (46.7 to 71.6)	61.3 (48.3 to 71.2)
**Sex**				
Male	217 (41.2%)	55 (61.1%)	113 (35.5%)	49 (41.2%)
Female	310 (58.8%)	35 (38.9%)	205 (64.5%)	70 (58.8%)
**Ethnicity**				
White	523 (99.6%)	88 (100%)	316 (99.4%)	119 (100%)
Other	2 (0.4%)	0 (0%)	2 (0.6%)	0 (0%)
**Marital Status**				
Married/civil partners/cohabiting	340 (64.6%)	68 (76.4%)	196 (61.6%)	76 (63.9%)
Separated/divorced/widowed	127 (24.1%)	16 (18.0%)	80 (25.2%)	31 (26.1%)
Single/other	59 (11.2%)	5 (5.6%)	42 (13.2%)	12 (10.1%)
**Housing tenure**				
Owner/joint owner/family owned	433 (82.3%)	73 (82.0%)	262 (82.4%)	98 (82.4%)
Other	93 (17.7%)	16 (18.0%)	56 (17.6%)	21 (17.6%)
**Employment**				
Employed	252 (47.8%)	36 (40.4%)	155 (48.4%)	61 (51.7%)
Retired	238 (45.2%)	47 (52.8%)	144 (45.0%)	47 (39.8%)
Other	37 (7.0%)	6 (6.7%)	21 (6.6%)	10 (8.5%)
**Education level**				
Degree or above	235 (44.8%)	29 (32.6%)	153 (48.2%)	53 (44.9%)
Below degree level	172 (32.8%)	32 (36.0%)	102 (32.2%)	38 (32.2%)
Other	46 (8.8%)	8 (9.0%)	29 (9.2%)	9 (7.6%)
None (no formal qualifications)	71 (13.5%)	20 (22.5%)	33 (10.4%)	18 (15.3%)
**English deprivation quintile**				
1 (least deprived)	49 (9.4%)	17 (19.1%)	30 (9.4%)	2 (1.7%)
2	221 (42.2%)	38 (42.7%)	138 (43.4%)	45 (38.5%)
3	202 (38.6%)	25 (28.1%)	114 (35.9%)	63 (53.9%)
4 and 5**	52 (9.9%)	9 (10.1%)	36 (11.3%)	7(6.0%)
**Long-term illness**				
Yes	394 (76.5%)	69 (80.2%)	241 (77.0%)	84 (72.4%)
No	121 (23.5%)	17(19.8%)	72 (23.0%)	32 (27.6%)

Note: proportions calculated among those with known information on the characteristic of interest
*With known exposure status
**The two most deprived quintiles were combined due to small numbers

For peer review only

Appendix II. EQ-5D-5L health-related dimensions according to exposure to flooding

EQ-5D-5L dimension	Level	Exposure to flooding					
		All	Unaffected	Disrupted	Flooded (all)	Single flooded	Repeat flooded
		n (%)	n (%)	n (%)	n (%)	n (%)	n (%)
Mobility problems	No	391 (75.2%)	65 (73.9%)	240 (76.4%)	86 (74.6%)	41 (75.9%)	45 (70.3%)
	Slight	59 (11.3%)	11 (12.5%)	36 (11.5%)	11 (9.3%)	1 (1.9%)	10 (15.6%)
	Moderate	49 (9.4%)	8 (9.1%)	25 (8.0%)	16 (13.6%)	10 (18.5%)	6 (9.4%)
	Severe/unable*	22 (4.2%)	4 (4.5%)	13 (4.1%)	5 (4.2%)	2 (3.7%)	3 (4.7%)
Self-care problems	No	482 (92.7%)	84 (95.5%)	290 (93.0%)	108 (91.5%)	49 (90.7%)	59 (92.2%)
	Slight	18 (3.5%)	2 (2.3%)	13 (4.1%)	3 (2.5%)	1 (1.9%)	2 (3.1%)
	Moderate	16 (3.1%)	2 (2.3%)	8 (2.5%)	6 (5.1%)	4 (7.4%)	2 (3.1%)
	Severe/unable*	4 (0.8%)	0 (0%)	3 (1.0%)	1 (0.8%)	0 (0%)	1 (1.6%)
Activity problems	No	403 (77.5%)	71 (80.7%)	247 (78.7%)	85 (72.0%)	37 (68.5%)	48 (75.0%)
	Slight	55 (10.6%)	9 (10.2%)	32 (10.2%)	14 (11.9%)	7 (13.0%)	7 (10.9%)
	Moderate	48 (9.2%)	7 (8.0%)	28 (8.9%)	13 (11.0%)	8 (14.8%)	5 (7.8%)
	Severe/unable*	14 (2.7%)	1 (1.1%)	7 (2.2%)	6 (5.1%)	2 (3.7%)	4 (6.3%)
Pain/discomfort	No	311 (59.8%)	51 (58.0%)	194 (61.8%)	66 (55.9%)	29 (53.7%)	37 (57.8%)
	Slight	122 (23.5%)	25 (28.4%)	67 (21.3%)	30 (25.4%)	15 (28.8%)	15 (23.4%)
	Moderate	63 (12.1%)	8 (9.1%)	39 (12.4%)	16 (13.6%)	7 (13.0%)	9 (14.1%)
	Severe/extreme*	24 (4.6%)	4 (4.5%)	14 (4.5%)	6 (5.1%)	3 (5.6%)	3 (4.7%)
Anxiety/depression	No	347 (66.7%)	71 (80.7%)	232 (73.9%)	44 (37.3%)	19 (35.2%)	25 (39.1%)
	Slight	96 (18.5%)	13 (14.8%)	53 (16.9%)	30 (25.4%)	18 (33.3%)	12 (18.8%)
	Moderate	60 (11.5%)	3 (3.4%)	23 (7.3%)	34 (28.8%)	14 (25.9%)	20 (31.3%)
	Severe/extreme*	17 (3.3%)	1 (1.1%)	6 (1.9%)	10 (8.5%)	3 (5.6%)	7 (10.9%)

*Groups combined due to small numbers

For peer review only

STROBE Statement—Checklist of items that should be included in reports of ***cross-sectional studies***

	Item No	Recommendation
**Title and abstract**	1	(*a*) Indicate the study's design with a commonly used term in the title or the abstract – p.2
		(*b*) Provide in the abstract an informative and balanced summary of what was done and what was found – p.2
**Introduction**		
Background/rationale	2	Explain the scientific background and rationale for the investigation being reported - p.4
Objectives	3	State specific objectives, including any prespecified hypotheses – p.4
**Methods**		
Study design	4	Present key elements of study design early in the paper – p.5-6
Setting	5	Describe the setting, locations, and relevant dates, including periods of recruitment, exposure, follow-up, and data collection – p.5-6
Participants	6	(*a*) Give the eligibility criteria, and the sources and methods of selection of participants – p.5
Variables	7	Clearly define all outcomes, exposures, predictors, potential confounders, and effect modifiers. Give diagnostic criteria, if applicable – p.5-6
Data sources/ measurement	8*	For each variable of interest, give sources of data and details of methods of assessment (measurement). Describe comparability of assessment methods if there is more than one group – p.5-6
Bias	9	Describe any efforts to address potential sources of bias – p.5
Study size	10	Explain how the study size was arrived at – p.5
Quantitative variables	11	Explain how quantitative variables were handled in the analyses. If applicable, describe which groupings were chosen and why – p.5-6
Statistical methods	12	(*a*) Describe all statistical methods, including those used to control for confounding – p.6
		(*b*) Describe any methods used to examine subgroups and interactions – p.6
		(*c*) Explain how missing data were addressed – p.6
		(*d*) If applicable, describe analytical methods taking account of sampling strategy N/A
		(*e*) Describe any sensitivity analyses – N/A
**Results**		
Participants	13*	(a) Report numbers of individuals at each stage of study—eg numbers potentially eligible, examined for eligibility, confirmed eligible, included in the study, completing follow-up, and analysed – p.6-7
		(b) Give reasons for non-participation at each stage – N/A
		(c) Consider use of a flow diagram – not necessary
Descriptive data	14*	(a) Give characteristics of study participants (eg demographic, clinical, social) and information on exposures and potential confounders – p.6-7 and Appendix I
		(b) Indicate number of participants with missing data for each variable of interest – p.6-7
Outcome data	15*	Report numbers of outcome events or summary measures – p.6-7
Main results	16	(*a*) Give unadjusted estimates and, if applicable, confounder-adjusted estimates and their precision (eg, 95% confidence interval). Make clear which confounders were adjusted for and why they were included – p.7 and Tables 1, 2, 3 and 4

For peer review only - http://bmjopen.bmj.com/site/about/guidelines.xhtml

		(*b*) Report category boundaries when continuous variables were categorized – p.5
		(*c*) If relevant, consider translating estimates of relative risk into absolute risk for a meaningful time period – N/A
Other analyses	17	Report other analyses done—eg analyses of subgroups and interactions, and sensitivity analyses – N/A
**Discussion**		
Key results	18	Summarise key results with reference to study objectives – p.8
Limitations	19	Discuss limitations of the study, taking into account sources of potential bias or imprecision. Discuss both direction and magnitude of any potential bias – p.8-9
Interpretation	20	Give a cautious overall interpretation of results considering objectives, limitations, multiplicity of analyses, results from similar studies, and other relevant evidence – p8-9
Generalisability	21	Discuss the generalisability (external validity) of the study results – p.8-9
**Other information**		
Funding	22	Give the source of funding and the role of the funders for the present study and, if applicable, for the original study on which the present article is based – p.11

*Give information separately for exposed and unexposed groups.

Note: An Explanation and Elaboration article discusses each checklist item and gives methodological background and published examples of transparent reporting. The STROBE checklist is best used in conjunction with this article (freely available on the Web sites of PLoS Medicine at http://www.plosmedicine.org/, Annals of Internal Medicine at http://www.annals.org/, and Epidemiology at http://www.epidem.com/). Information on the STROBE Initiative is available at www.strobe-statement.org.

For peer review only

BMJ Open

Impact of repeat flooding on mental health and health-related quality of life: a cross-sectional analysis of the English National Study of Flooding and Health

Journal:	*BMJ Open*
Manuscript ID	bmjopen-2019-031562.R2
Article Type:	Original research
Date Submitted by the Author:	05-Sep-2019
Complete List of Authors:	French, Clare; University of Bristol, Population Health Sciences, Bristol Medical School<br>Waite, Thomas; Public Health England, National Infection Service<br>Armstrong, Ben; London School of Hygiene and Tropical Medicine<br>Rubin, GJ; King's College London, NIHR Health Protection Research Unit in Emergency Preparedness and Response, Department of Psychological Medicine, Weston Education Centre<br>National Study of Flooding and Health Study Group, English ; Public Health England<br>Beck, Charles; Public Health England, Field Service South West, National Infection Service; Bristol Medical School, NIHR Health Protection Unit in Evaluation of Interventions, Population Health Sciences<br>Oliver, Isabel; Public Health England, Field Service South West, National Infection Service; Bristol Medical School, NIHR Health Protection Unit in Evaluation of Interventions, Population Health Sciences
**Primary Subject Heading**:	Epidemiology
Secondary Subject Heading:	Mental health, Public health
Keywords:	EPIDEMIOLOGY, PUBLIC HEALTH, MENTAL HEALTH, Health-related quality of life, Flooding

SCHOLARONE™
Manuscripts

Impact of repeat flooding on mental health and health-related quality of life: a cross-sectional analysis of the English National Study of Flooding and Health

Clare E French[1], Thomas D Waite[2], Ben Armstrong[3], G. James Rubin[4], English National Study of Flooding and Health Study Group, Charles R Beck[1,5] Isabel Oliver[1,5]

[1] NIHR Health Protection Unit in Evaluation of Interventions, Population Health Sciences, Bristol Medical School, University of Bristol, BS8 2PS, UK

[2] National Infection Service, Public Health England, London, UK

[3] NIHR Health Protection Research Unit in Environmental Change and Health at the London School of Hygiene and Tropical Medicine, London, UK

[4] Department of Psychological Medicine, Weston Education Centre, King's College London NIHR Health Protection Research Unit in Emergency Preparedness and Response, London, UK

[5] Field Service South West, National Infection Service, Public Health England, Bristol, UK

Corresponding author: Clare French, Population Health Sciences, Bristol Medical School, Canynge Hall, 39 Whatley Road, Bristol BS8 2PS, UK. Email: clare.french@bristol.ac.uk. Tel: +44(0)117 3313925

Word count: 3,272

For peer review only - http://bmjopen.bmj.com/site/about/guidelines.xhtml

ABSTRACT

Objective To assess the association between flooding/repeat flooding and: i) psychological morbidity (anxiety, depression, post-traumatic stress disorder [PTSD]) and ii) health-related quality of life (HRQoL) at six months post-flooding.

Design Cross-sectional analysis of data from the English National Study of Flooding and Health.

Setting Cumbria, England.

Participants Questionnaires were sent to 2500 residential addresses at six months post-flooding; 590 people responded.

Outcomes Probable depression was assessed using the Patient Health Questionnaire (PHQ-2); probable anxiety using the Generalised Anxiety Disorder scale (GAD-2); and probable PTSD using the short-form PTSD checklist (PCL-6). Health-related quality of life (HRQoL) was assessed using the EQ-5D-5L.

Mental health outcomes were analysed using logistic regression; HRQoL dimensions using ordinal regression; and summary index/VAS scores using linear regression.

Results One hundred and nineteen participants had been flooded, over half of whom were experiencing a repeat flooding event (54%; n=64).

Mental health outcomes were elevated among flooded compared with unaffected participants (adjusted odds ratio for probable depression: 7.77, 95%CI: 1.51 to 40.13; anxiety: 4.16, 95%CI: 1.18 to 14.70; PTSD: 14.41, 95%CI: 3.91 to 53.13). The prevalence of depression was higher among repeat compared with single flooded participants, but this was not significant after adjustment. There was no difference in levels of anxiety or PTSD.

Compared with unaffected participants, those flooded had lower EQ-5D-5L index scores (adjusted coefficient: -0.06, 95% CI: -0.12 to -0.01) and lower self-rated health scores (adjusted coefficient: -6.99, 95% CI: -11.96 to -2.02). There was, however, little difference in HRQoL overall between repeat and single flooded participants.

Conclusions Interventions are needed to help minimise the impact of flooding on people’s mental health and HRQoL.

For peer review only

Strengths and limitations of this study

- There is currently very little research exploring the impact of repeat flooding on mental health or health-related quality of life – our research addresses a gap in the evidence
- We use data from the English National Study of Flooding and Health – a large, robust study with proven methodology
- Three key mental health outcomes (depression, anxiety, post-traumatic stress disorder) as well as health-related quality of life were assessed, all using validated instruments
- The tools used to assess psychological outcomes are indictive of probable diagnoses rather than being clinically diagnostic
- Information was not available on the number or timing previous flooding events

For peer review only

For peer review only - http://bmjopen.bmj.com/site/about/guidelines.xhtml

INTRODUCTION

Flooding can have a range of effects on people's health, both physical and psychological.[1-5] Given projections that both the frequency and severity of flooding events will continue to increase in the future,[6-9] populations living in flood-susceptible areas may be exposed to multiple flooding events. It is important that the impact of flooding, and repeat flooding, on people's health and wellbeing is thoroughly understood. This will help inform public health actions to mitigate adverse health consequences of future flooding events.

Beyond the acute health consequences of flooding, such as injuries and infections, the longer-term health consequences are thought to be primarily psychological.[6] The English National Study of Flooding and Health began in 2015 to assess the impact of widespread flooding in southern England. The study has assessed the impact of flooding and related disruption on mental health and wellbeing over several years. Data obtained one year after flooding in southern England showed higher levels of depression, anxiety and post-traumatic stress disorder (PTSD) in participants whose homes had been flooded, and to a lesser extent, those whose lives had been disrupted by flooding compared to those who were unaffected.[10] Follow-up of participants at two years post-flooding demonstrated that mental health consequences had waned but were still noticeably higher in affected groups.[11] The impact of flooding on health-related quality of life (HRQoL) has not yet been reported on.

Several factors are known to be associated with the likelihood of experiencing adverse mental health outcomes post-flooding. These include socio-demographic characteristics such as age, gender, socio-economic and education statuses, and factors related to the nature of flooding e.g. depth and duration.[12] Secondary stressors, including problems with relationships, loss of sentimental items, and difficulties with insurance or compensation have been associated with poor psychological outcomes.[13, 14]. Meanwhile, societal-level factors such as social cohesion and support, as well as individual-level factors such as positive/proactive coping strategies can be protective against the development of mental ill health in the aftermath of flooding.[2,15] Although there is less published research on the association between flooding and wellbeing/quality of life, the available evidence indicates that effects similarly occur through a complex set of inter-related pathways.[16]

The UK Climate Change Risk Assessment 2017 highlighted flooding as a risk to people, communities and buildings that requires more action.[17] More research is required to assess and understand how these risks are best tackled. There is currently very limited research exploring the impact of experiencing repeated flooding events on mental health and/or HRQoL, and the evidence to-date is inconclusive.[15,18-20]

In early December 2015, exceptionally heavy and persistent rainfall associated with storm "Desmond", resulted in widespread flooding in Cumbria and other parts of northern Britain. Thousands of homes and businesses across Cumbria were flooded, and tens of thousands left without power.[21,22] Cumbria is an area that has been repeatedly affected by flooding - the region had experienced severe flooding just six years previously in 2009, and also in 2005.[22]

The objective of this study is to assess the association between flooding/repeat flooding and: i) psychological morbidity (probable anxiety, probable depression, probable PTSD) and ii) HRQoL, in the Cumbria region at six months post-flooding.

For peer review only - http://bmjopen.bmj.com/site/about/guidelines.xhtml

METHODS

Study conduct

Details on the design and conduct of the English National Study of Flooding and Health have been published previously.[10] Recruitment and data collection in Cumbria largely mirrored the original survey conducted elsewhere. Recruitment packs (including consent forms, study information sheets and questionnaires) were sent to 2500 residential households in postcode areas known to have been affected by the 2015/16 floods within four local authority areas (Allerdale, Carlisle, Eden, South Lakeland). The sample size was determined with the aim of sampling approximately 50% of flooded households. All adults in each household were invited to participate. Packs were sent out approximately six months post-flooding (May 2016), with two rounds of reminder letters sent to non-responders. Invitations were sent by post and participants had the option to respond either by post or online. Questionnaires were similar to those used for the original survey, with some minor clarification to the wording of questions, and the addition of the EQ-5D-5L instrument to assess HRQoL.

Participants were asked about their exposure to the 2015/16 floods, and whether they had been previously flooded. They were categorised as:

- Unaffected - those who experienced no flooding or disruption during the 2015/16 floods
- Disrupted - those who did not have floodwater in liveable areas of their homes but reported experiencing disruption due to the floods (e.g. floodwater in non-liveable areas such as a garage, disruption to loss of utilities, loss of access to services)
- Flooded - those who reported having floodwater in one or more liveable rooms of their home (e.g. living room, kitchen, bedroom). For analyses of repeat flooding this group was further sub-categorised into:
    - Repeat flooded - those who reported having been previously flooded (either in the same or a previous home),
    - Single flooded - those who reported not having been previously flooded.

Demographic information collected included date of birth, sex, ethnicity, long-term illness or disability, education, employment and marital statuses. Postcodes of residence were used to assign Lower Super Output Area deprivation scores based on the Index of Multiple Deprivation.[23] These were ranked according to scores for all of England and categorised into quintiles.

Mental health outcomes were assessed using validated instruments - probable depression using the Patient Health Questionnaire (PHQ-2) depression sub-scale; probable anxiety using the Generalised Anxiety Disorder scale (GAD-2); and probable PTSD using the short-form PTSD checklist (PCL-6). Cut-off scores, used to indicate the likely presence of the condition, were ≥3 for PHQ-2/GAD-2 and ≥14 for PCL-6.[24-26] We also generated a combined variable indicating the presence of 'any' of these probable conditions.

HRQoL was assessed using the EQ-5D-5L. This is a validated two-part instrument. The first part is a descriptive system assessing five health-related dimensions (mobility, self-care, usual activities, pain/discomfort and anxiety/depression) on a five-point scale (no problems, slight problems, moderate problems, severe problems and extreme problems). Information on these five health states was converted into a single index value. The second part is a visual analogue scale (VAS) which records the patient's self-rated health on a vertical scale from marked from 0

For peer review only - http://bmjopen.bmj.com/site/about/guidelines.xhtml

to 100 where the endpoints are ‘The best health you can imagine’ and ‘The worst health you can imagine’.[27]

SelectSurvey (ClassApps, USA) was used to develop the online questionnaire and capture online responses. Data returned on paper forms were double-entered using EpiData (EpiData Association, Denmark) and cross-checked to identify any errors.

Data analysis

Data were analysed in STATA 13.1 (StataCorp, USA). Participants who did not provide enough information to enable an exposure status to be assigned to them were excluded from analyses by exposure group. For analyses of mental health outcomes, participants who did not complete an outcome instrument were excluded from analyses of that outcome only. HRQoL analyses were restricted to those with complete information on both parts of the tool.

Mental health outcomes were analysed using binary logistic regression. For analyses of HRQoL, the EQ-5D-5L descriptive system ordered categorical variables were analysed using ordinal logistic regression. The proportional odds assumption was checked for each outcome using approximate likelihood-ratio tests. Continuous variables (EQ-5D-5L index score and VAS) both had left-skewed distributions and are thus presented as medians and inter-quartile ranges (IQRs). Medians were compared using the Mann Whitney U test and adjusted analyses were conducted using linear regression with robust standard errors to allow for the skewed distribution of the dependent variable.

Potential socio-demographic confounders of the association between flooding and mental health outcomes have been previously identified (age group, sex, local authority, ethnicity, marital status, education level, employment, deprivation score and pre-existing illness).[10] All (except ethnicity, due to very small numbers in the non-white ethnic group) were adjusted for *a priori* in all multivariable analyses for consistency and comparability between models.

Patient and public involvement

A community flooding action group was involved in the design of the study. Volunteers identified by the National Flood Forum piloted early versions of the questionnaires, and the National Flood Forum Chief Executive is a member of our stakeholder group. Participants were invited to join a study group to support the project and keep track of its progress. Study findings are being disseminated to participants via reports published online.

RESULTS

Response rates

Completed questionnaires were received from 590 participants (household response rate: 24.4%). More than one individual responded from 19 households giving a total of 628 participating individuals. Most participants responded using the paper questionnaire, with 7.0% (n=44) responding online. Subsequent analyses were conducted on the 531 participants who provided sufficient information to enable an exposure status to be assigned to them.

Participant characteristics

The socio-demographic characteristics of study participants are provided in Appendix I. Overall, the median age was 62 years (inter-quartile range [IQR]: 49 to 72 years), 58.8% were female,

For peer review only - http://bmjopen.bmj.com/site/about/guidelines.xhtml
For peer review only

99.6% were of white ethnicity, 51.6% lived in areas belonging to the two least deprived quintiles and 76.5% had a long-term illness or disability.

Exposure to flooding and psychological morbidity

Overall, 16.9% of participants were unaffected by the floods, 60.6% were disrupted and 22.4% were flooded.

After adjusting for socio-demographic factors, there was an elevated odds of each mental health outcome among flooded participants compared to those unaffected (adjusted odds ratio [aOR] for probable depression: 7.77, 95% confidence interval [CI]: 1.51 to 40.13; anxiety: 4.16, 95% CI: 1.18 to 14.70; PTSD: 14.41, 95% CI: 3.91 to 53.13; and for 'any' of these mental health outcomes: 11.81, 95% CI: 3.99-34.96). The adjusted odds of probable depression, PTSD and any outcome (but not anxiety) were also somewhat raised for those who experienced disruption due to flooding but not significantly so (Table 1).

Of those flooded during the 2015/16 floods, over half (54%; 64/119) had been previously flooded (60 at the same home and four at a previous home). The prevalence of probable depression was elevated among those exposed to repeated, as compared with a single flooding event, but the difference reduced on adjustment for potential confounders, and was not significant. For probable anxiety and PTSD there was little difference in prevalence among people who experienced single and repeat flooding, either before or after adjustment (Table 1).

Exposure to flooding and HRQoL

Most participants (97.9%, n=520) had complete information on both parts of the EQ-5D-5L.

Index scores

Median EQ-5D-5L index scores are presented in Table 2. In adjusted analyses flooded participants (all) had lower EQ-5D-5L index scores than unaffected participants (adjusted coefficient: -0.06, 95% CI: -0.12 to -0.01). There was little evidence that scores differed between repeat and single flooded participants.

Health-related dimensions

The level of problems reported by participants on each dimension, according to exposure to flooding are provided in Appendix II.

Flooded participants were more likely to have higher anxiety/depression scores than those unaffected (aOR: 8.59, 95% CI:4.02 to 18.36; Table 3), also more self-care and activity problems, though not significantly so. There was little evidence that the flooded group had more mobility problems or pain/discomfort than those unaffected. There was little evidence of a difference between repeat and single flooded participants on any dimension (Table 3). There was no evidence against the proportional odds assumption for any of the five dimensions.

VAS scores

Median VAS scores are presented in Table 4. Scores were 78 (IQR 69 to 90) for flooded participants, notably lower than for those unaffected (90, IQR: 75 to 93). This difference remained after adjustment (adjusted coefficient of VAS scores among flooded vs. unaffected participants: -6.99, 95% CI: -11.96 to -2.02). There was little evidence of a difference in VAS scores between the repeat and single flooded groups. For analyses of both index scores and

For peer review only - http://bmjopen.bmj.com/site/about/guidelines.xhtml

VAS scores the key assumptions of linear regression were met; robust standard errors were used which dealt with the non-normality of the data.

DISCUSSION

This study provides strong evidence of an association between flooding, be it a single or repeat episode, and probable mental health outcomes. Flooded participants also tended to have poorer HRQoL than those unaffected by the floods. Analyses of the dimension-specific descriptive EQ-5D-5L system indicate that anxiety/depression may be an important driver of this association. There was little evidence of a difference in either probable mental health outcomes or HRQoL between repeat and single flooded participants, although data were suggestive of a greater level of depression in those repeatedly flooded.

The UK Climate Change Risk Assessment 2017 identifies flooding as a major risk to people and the built environment; more action is needed to mitigate this risk.[17] The UK government has committed to actions, such as ensuring that people have good access to information to assess the risks, including health risks, of flooding; helping people to make their properties more resistant to flooding; and ensuring plans are in place to predict and respond to flooding incidents.[28] Our research should be used to inform that government action - although we focus on the impact of flooding on health and wellbeing, the evidence presented here supports the case for good town planning, flood and coastal risk management, and the implementation of the 25-year Environment Plan.[28]

The overall associations between being flooded (vs. unaffected) and mental health outcomes were consistent with those measured in the cohort affected by the floods in the winter of 2013/14 in southern England.[10] Those floods largely affected affluent areas potentially limiting the generalisability of the findings beyond that population. Participants from Cumbria resided in areas of comparatively greater deprivation; our findings thus demonstrate that the increased risks of mental health outcomes following flooding also pertain to those living in more deprived areas. This is consistent with low income having been previously reported as a known driver of vulnerability to the effects of flooding.[29] Of note, a strong association between flooding and anxiety/depression was found using the EQ-5D-5L, corroborating findings obtained using mental health outcome assessment tools.

There is limited previous research on the impact of flooding on HRQoL with which to draw comparisons, and we are not aware of any previously published comparable data on the impact of repeat flooding specifically. A mixed-methods study in two UK regions found that flooding had a marked impact on wellbeing. This occurred via a complex and diverse range of interacting factors acting at both the individual and societal level.[16] Data from China showed poorer quality of life among those affected by floods compared with those unaffected, but the EQ-5D-5L tool was not used and the findings are not directly comparable.[30]

That over half of flooded participants in our sample had experienced repeat flooding is striking. It may be hypothesized that this group will suffer greater mental health and HRQoL effects than those experiencing single event due to the cumulative effects of these experiences. However, our research indicates that any association may be more nuanced. The data were suggestive of higher levels of depression, but not other mental health outcomes or overall HRQoL, among repeat compared with single flooded participants. Of note, the number of events were small in some groups resulting in imprecise estimates – the data should thus be interpreted with some caution. The lack of association found in our study does, however, concur with a recently

For peer review only - http://bmjopen.bmj.com/site/about/guidelines.xhtml

published Australian study which reported a general lack of association between exposure to repeat natural disasters and the broader psychiatric disorder spectrum.[31]

Repeat flooding may impact on mental health in a variety of ways, though the evidence is inconclusive.[15,18,19] At the individual level, experiencing a repeat episode of flooding may trigger memories of previous flooding episodes, intensifying the distress experienced.[18] Those who have been previously flooded may also experience anticipatory anxiety and stress about the prospect of future flooding.[32,33] Conversely, people's individual and collective coping capacity may be increased, and they may display greater resilience to future flooding events.[34] There is also evidence to suggest that previous exposure to disasters may have an 'inoculation' effect, particularly among older adults (the median age of our study population was 62 years).[20, 35, 36] Meanwhile, factors acting at the community level may mitigate the risks of adverse consequences of future floods through the development or strengthening of community networks and increased social cohesion.[16,32,37] Finally, past flood events may have resulted in improvements to infrastructure, flood defences and response systems, reducing the extent of subsequent floods and the impact on people's lives.

General limitations of the National Study of Flooding and Health have been outlined elsewhere.[10] Our questionnaire response rate of 24% is in line with other similar postal surveys.[38,39] Although this relatively low response rate is a potential limitation of our study, it would only bias the observed associations between flooding and mental health / HRQoL if response was differential with respect to both flooding and health outcomes. Although this is a possibility, we don't have evidence to suggest that any such differential response would be substantial. Another limitation of these analyses specifically is that we do not have any information on the number, timing or extent of previous flooding experiences. Such information would be helpful in interpreting our findings and should be considered when designing future research studies on the health impacts of flooding.

Though validated instruments, the tools used to assess psychological outcomes are not clinically diagnostic. Our data therefore indicate 'probable' diagnoses which are likely an overestimate of diagnosable mental health conditions. Meanwhile, the EQ-5D-5L is a validated and widely used tool that has been previously used in UK populations.[40] The tool was well completed (98% of participants had complete data) indicating its acceptability in our study. However, some of the dimensions assessed may not be directly relevant to the types of illnesses that may occur as a consequence of flooding. At the same time, due to space constraints on the questionnaire, we were unable to assess all psychological variables that are of theoretical interest following repeat flooding. For example, anger might be elevated in flooded, and especially repeat flooded, participants and may be worthy of future investigation.

Our findings may not be generalisable to all populations affected by flooding (e.g. there were few participants of non-white ethnicity). However, all comparison groups were drawn from the same population and key socio-demographic confounding factors were adjusted for in the analyses.

Conclusions and recommendations

Flooding can have a profound effect on people's psychological health, and aspects of their HRQoL. Though we found little evidence that people exposed to repeat flooding were at increased risk of adverse outcomes compared with those exposed to a single event, it should be remembered that these individuals are at risk of experiencing adverse outcomes each time they experience flooding.

For peer review only - http://bmjopen.bmj.com/site/about/guidelines.xhtml

That over half of participants in this study were experiencing a repeat flooding event highlights the need for strengthening flood defences and mitigating risks of future flooding in areas at risk or previously affected. The UK's National Adaptation Programme for climate change recommends actions for reducing flood risk and harm from flooding including effective land use planning;[28] the evaluation of these planned actions should include an assessment of their impact on people's mental health and wellbeing.

Interventions are needed to help minimise the impact of flooding on mental health and wellbeing. These may include early warning systems which can reduce the effects of flooding on mental health,[41] and community-based psychosocial support such as psychological first aid in the immediate aftermath of flood events. Further research on the effectiveness of these and other interventions to reduce the impact of flooding on people's health would be informative in shaping future intervention strategies.

Providing people with other approaches to reduce their susceptibility to the effects of flooding, such as efforts to increase community cohesion and develop support networks in preparedness for potential future flooding events, may also be beneficial. Finally, ensuring that adequate and effective mental health services are available for community members following flooding is vital.

For peer review only

Acknowledgments

We would like to thank the participants in this study and are grateful for the support we received from Colin Cox, Director of Public Health Cumbria County Council and the North West Public Health England Centre. We acknowledge the role of other members of the English National Study of Flooding and Health Study Group in the design of the National Study of Flooding and Health: Katerina Chaintarli, Angie Bone, Richard Amlôt, Sari Kovats, Mark Reacher and Giovanni Leonardi. We would also like to thank all PHE staff who contributed to study administration, set up and data entry.

Part of this work was presented at the Public Heath England Public Health Research and Science Conference 2018, Warwick, UK.

Ethics approval

Ethical approval for the study was granted by the Psychiatry, Nursing and Midwifery Research Ethics Subcommittee at King's College London [Reference PNM 1314 152]. All participants consented to participate in the study.

Data sharing

The datasets analysed in this study are available from Public Health England Field Epidemiology Service on reasonable request.

Competing interests

The authors declare that they have no competing interests.

Funding

The research was funded in part by the National Institute for Health Research Health Protection Research Units (NIHR HPRU) in Emergency Preparedness and Response at King's College London, Environmental Change at the London School of Hygiene and Tropical Medicine and Evaluation of Interventions at the University of Bristol, in partnership with Public Health England (PHE). The views expressed are those of the authors and not those of the NHS, the NIHR, The Department of Health or Public Health England.

Author contributions

CEF led the design and conduct of these analyses, with expert statistical advice from BA, and drafted the manuscript. English National Study of Flooding and Health Study Group members including named authors IO, TDW, BA, CRB and GJR designed the National Study of Flooding and Health and acquired the data. IO, TDW, BA, CRB and GJR contributed to the design of the analyses and the interpretation the findings. All authors contributed to the writing of the manuscript and approved it for submission.

Exclusive license

The Submitting Author accepts and understands that any supply made under these terms is made by BMJ to the Submitting Author unless you are acting as an employee on behalf of your employer or a postgraduate student of an affiliated institution which is paying any applicable article publishing charge ("APC") for Open Access articles. Where the Submitting Author wishes to make the Work available on an Open Access basis (and intends to pay the relevant APC), the terms of reuse of such Open Access shall be governed by a Creative Commons licence – details of these licences and which Creative Commons licence will apply to this Work are set out in our licence referred to above.

For peer review only - http://bmjopen.bmj.com/site/about/guidelines.xhtml

Table 1. Prevalence and odds ratios (crude and adjusted) of mental health outcomes according to exposure to flooding

	n/N (%)*	OR (95% CI)	aOR** † (95% CI)
**MODEL 1 - Comparing flooded and disrupted with unaffected participants**			
**Probable depression**			
Unaffected	2/83 (2.4%)	Ref	Ref
Disrupted	15/292 (5.1%)	2.19 (0.49 to 9.80)	1.38 (0.27 to 6.96)
Flooded	25/106 (23.6%)	12.50 (2.87 to 54.52)	7.77 (1.51 to 40.13)
**Probable anxiety**			
Unaffected	5/83 (6.0%)	Ref	Ref
Disrupted	19/289 (6.6%)	1.10 (0.40 to 3.03)	0.91 (0.27 to 3.08)
Flooded	24/107 (22.4%)	4.51 (1.64 to 12.41)	4.16 (1.18 to 14.70)
**Probable PTSD**			
Unaffected	3/87 (3.4%)	Ref	Ref
Disrupted	30/300 (10.0%)	3.11 (0.93 to 10.45)	2.05 (0.57 to 7.40)
Flooded	49/112 (43.8%)	21.78 (6.49 to 73.08)	14.41 (3.91 to 53.13)
**'Any' probable mental health outcome**			
Unaffected	6/82 (7.3%)	Ref	Ref
Disrupted	41/285 (14.4%)	2.13 (0.87 to 5.21)	1.88 (0.67 to 5.32)
Flooded	52/107 (48.6%)	11.98 (4.08 to 29.86)	11.81 (3.99 to 34.96)
**MODEL 2 - Comparing repeat with single flooded participants**			
**Probable depression**			
Single flooded	8/49 (16.3%)	Ref	Ref
Repeat flooded	17/57 (29.8%)	2.18 (0.85 to 5.61)	1.55 (0.44 to 5.46)
**Probable anxiety**			
Single flooded	11/50 (22.0%)	Ref	Ref
Repeat flooded	13/57 (22.8%)	1.05 (0.42 to 2.61)	0.89 (0.26 to 2.97)
**Probable PTSD**			
Single flooded	21/51 (41.2%)	Ref	Ref
Repeat flooded	28/61 (45.9%)	1.21 (0.57 to 2.57)	0.73 (0.27 to 1.97)
**'Any' probable mental health outcome**			
Single flooded	22/48 (45.8%)	Ref	Ref
Repeat flooded	30/59 (50.8%)	1.22 (0.57 to 2.62)	1.03 (0.39 to 2.73)

OR – odds ratio, aOR – adjusted odds ratio, CI – confidence interval

*N is the total number of participants in each exposure group for whom a complete answer to this outcome instrument was provided, and % is the proportion of those who have the relevant (probable) condition

For peer review only

For peer review only - http://bmjopen.bmj.com/site/about/guidelines.xhtml

**Adjusted for age group, sex, local authority, marital status, education level, employment, deprivation score and pre-existing illness

†Number of observations included in the multivariable models were: probable depression n=446, probable anxiety n=444, probable PTSD n=464, 'any' probable mental health outcome n=440. Both models included the same participants but the reference group differs. Odds ratios are only presented for the comparison of interest

For peer review only

For peer review only - http://bmjopen.bmj.com/site/about/guidelines.xhtml

Table 2. Univariable and multivariable analyses of the association between exposure to flooding and EQ-5D-5L index values

	Exposure group	Median (IQR) index value	Crude coefficient (95% CI)*	Adjusted coefficient (95% CI)* **
MODEL 1	Unaffected	1.00 (0.77 to 1.00)	Ref	Ref
	Disrupted	1.00 (0.77 to 1.00)	-0.00 (-0.52 to 0.04)	-0.00 (-0.44 to 0.04)
	Flooded	0.85 (0.69 to 1.00)	-0.08 (-0.14 to -0.02)	-0.06 (-0.12 to -0.01)
MODEL 2	Single flooded	0.84 (0.68 to 0.88)	Ref	Ref
	Repeat flooded	0.85 (0.69 to 1.00)	0.00 (-0.08 to 0.08)	0.02 (-0.05 to 0.09)

*Confidence intervals estimated using robust standard errors

**Adjusted for age group, sex, local authority, marital status, education level, employment, deprivation score and pre-existing illness

For peer review only - http://bmjopen.bmj.com/site/about/guidelines.xhtml

Table 3. Univariable and multivariable analyses of EQ-5D-5L health-related dimensions according to exposure to flooding

	OR (95% CI)	aOR (95% CI)* **
**MODEL 1 - Comparing flooded and disrupted with unaffected participants**		
**Mobility problems**		
Unaffected	Ref	Ref
Disrupted	0.87 (0.51 to 1.49)	0.98 (0.48 to 2.02)
Flooded	1.09 (0.59 to 2.02)	1.04 (0.44 to 2.46)
**Self-care problems†**		
Unaffected	Ref	Ref
Disrupted	1.73 (0.58 to 5.12)	1.61 (0.41 to 6.31)
Flooded	1.98 (0.60 to 6.52)	1.62 (0.33 to 7.91)
**Activity problems**		
Unaffected	Ref	Ref
Disrupted	1.15 (0.64 to 2.07)	1.59 (0.70 to 3.60)
Flooded	1.68 (0.87 to 3.25)	1.85 (0.72 to 4.79)
**Pain/discomfort**		
Unaffected	Ref	Ref
Disrupted	0.92 (0.58 to 1.46)	0.95 (0.54 to 1.68)
Flooded	1.14 (0.67 to 1.95)	1.01 (0.50 to 2.02)
**Anxiety/depression**		
Unaffected	Ref	Ref
Disrupted	1.51 (0.84 to 2.70)	1.46 (0.73 to 2.91)
Flooded	7.72 (4.11 to 14.50)	8.59 (4.02 to 18.36)
**MODEL 2 - Comparing repeat with single flooded participants**		
**Mobility problems**		
Single flooded	Ref	Ref
Repeat flooded	1.16 (0.52 to 2.63)	0.85 (0.28 to 2.54)
**Self-care problems†**		
Single flooded	Ref	Ref
Repeat flooded	0.82 (0.23 to 3.01)	0.42 (0.08 to 2.29)
**Activity problems**		
Single flooded	Ref	Ref
Repeat flooded	0.74 (0.33 to 1.64)	0.71 (0.24 to 2.11)
**Pain/discomfort**		
Single flooded	Ref	Ref
Repeat flooded	0.88 (0.43 to 1.77)	0.52 (0.21 to 1.26)
**Anxiety/depression**		
Single flooded	Ref	Ref
Repeat flooded	1.20 (0.62 to 2.32)	1.13 (0.51 to 2.51)

For peer review only - http://bmjopen.bmj.com/site/about/guidelines.xhtml

For peer review only

*Adjusted for age group, sex, local authority, marital status, education level, employment, deprivation score and pre-existing illness

**Number of observations included in all multivariable models was 484. Both models 1 and 2 included the same participants, but the reference group differed. Odds ratios are only presented for the comparison of interest

†Due to very small numbers of participants with 'severe/extreme problems' on the self-care dimension, the 'severe/extreme' and 'moderate' categories were combined

For peer review only

For peer review only - http://bmjopen.bmj.com/site/about/guidelines.xhtml

Table 4. Univariable and multivariable analyses of the association between exposure to flooding and EQ-5D-5L VAS scores

	Exposure group	Median (IQR) VAS score	Crude coefficient (95% CI)*	Adjusted coefficient (95% CI)* **
MODEL 1	Unaffected	90 (75 to 93)	Ref	Ref
	Disrupted	85 (75 to 90)	-2.18 (-6.15 to 1.79)	-1.77 (-5.45 to 1.91)
	Flooded	78 (69 to 90)	-8.66 (-13.66 to -3.67)	-6.99 (-11.96 to -2.02)
MODEL 2	Single flooded	75 (70 to 85)	Ref	Ref
	Repeat flooded	80 (65 to 90)	-0.64 (-7.78 to 6.50)	0.76 (-7.06 to 8.60)

*Confidence intervals estimated using robust standard errors

**Adjusted for age group, sex, local authority, marital status, education level, employment, deprivation score and pre-existing illness

For peer review only

References

1. Ahern M, Kovats RS, Wilkinson P, et al. Global health impacts of floods: epidemiologic evidence. *Epidemiol Rev* 2005;27:36-46.

2. Fernandez A, Black J, Jones M, et al. Flooding and mental health: a systematic mapping review. *PLoS One* 2015;10(4):e0119929.

3. Saulnier DD, Brolin Ribacke K, von Schreeb J. No Calm After the Storm: A Systematic Review of Human Health Following Flood and Storm Disasters. *Prehosp Disaster Med* 2017;32(5):568-79.

4. Stanke C, Murray V, Amlot R, et al. The effects of flooding on mental health: Outcomes and recommendations from a review of the literature. *PLoS Currents* 2012; e4f9f1fa9c3cae. doi:10.1371/4f9f1fa9c3cae.

5. Zhong S, Yang L, Toloo S, et al. The long-term physical and psychological health impacts of flooding: A systematic mapping. *Sci Total Environ* 2018;626:165-94.

6. World Health Organization Regional Office for Europe. Floods in the WHO European Union: health effects and their prevention. Copenhagen, Denmark: World Health Organization, 2013:1-146. http://www.euro.who.int/__data/assets/pdf_file/0020/189020/e96853.pdf (Accessed September 2018).

7. European Environment Agency, European Commission Joint Research Centre and the World Health Organization. Impacts of Europe's changing climate - 2008 indicator-based assessment. Joint EEA-JRC-WHO report. Copenhagen, Denmark: European Environment Agency, European Commission Joint Research Centre and the World Health Organization, 2008:1-246. https://ec.europa.eu/jrc/sites/jrcsh/files/jrc_reference_report_2008_09_climate_change.pdf (Accessed September 2018).

8. Alfieri L, Bisselink B, Dottori F, et al. Global projections of river flood risk in a warmer world. *Earths Future* 2017;5(2):171-82.

9. Sayers PB, Horritt M, Penning-Rowsell E, et al. Climate Change Risk Assessment 2017: Projections of future flood risk in the UK. London, UK: Committee on Climate Change, 2017. https://www.theccc.org.uk/wp-content/uploads/2015/10/CCRA-Future-Flooding-Main-Report-Final-06Oct2015.pdf (Accessed October 2018).

10. Waite TD, Chaintarli K, Beck CR, et al. The English national cohort study of flooding and health: cross-sectional analysis of mental health outcomes at year one. *BMC Public Health* 2017;17(1):129.

11. Jermacane D, Waite TD, Beck CR, et al. The English National Cohort Study of Flooding and Health: the change in the prevalence of psychological morbidity at year two. *BMC Public Health* 2018;18(1):330.

12. Lowe D, Ebi KL, Forsberg B. Factors increasing vulnerability to health effects before, during and after floods. *Int J Environ Res Public Health* 2013;10(12):7015-67.

13. Tempest EL, English National Study on F, Health Study G, et al. Secondary stressors are associated with probable psychological morbidity after flooding: a cross-sectional analysis. *Eur J Public Health* 2017;27(6):1042-47.

14. Mulchandani R, Smith M, Armstrong B, English National Study of Flooding Health Study Group, Beck CR, Oliver I. Effect of Insurance-Related Factors on the Association between Flooding and Mental Health Outcomes. *Int J Environ Res Public Health* 2019;16(7):1174.

For peer review only - http://bmjopen.bmj.com/site/about/guidelines.xhtml

15. Greene G, Paranjothy S, Palmer SR. Resilience and Vulnerability to the Psychological Harm From Flooding: The Role of Social Cohesion. *Am J Public Health* 2015;105(9):1792-5.

16. Walker-Springett K, Butler C, Adger WN. Wellbeing in the aftermath of floods. *Health Place* 2017;43:66-74.

17. Committee on Climate Change. UK Climate Change Risk Assessment 2017. Synthesis report: priorities for the next five years. London, UK: Committee on Climate Change, 2016. https://www.theccc.org.uk/wp-content/uploads/2016/07/UK-CCRA-2017-Synthesis-Report-Committee-on-Climate-Change.pdf (Accessed October 2018).

18. Lamond J. The role of flood memory in the impact of repeat flooding on mental health. In: Proverbs D. and Brebbia, C., eds. *Flood Recovery, Innovation and Response IV*. Ashurst, UK: WIT Press, 2014. https://www.witpress.com/books/978-1-84564-784-1 (Accessed August 2018)

19. Mason V, Andrews H, Upton D. The psychological impact of exposure to floods. *Psychol Health Med* 2010;15(1):61-73.

20. Norris FH, Murrell SA. Prior experience as a moderator of disaster impact on anxiety symptoms in older adults. *Am J Community Psychol* 1988;16(5):665-83.

21. Met Office. Flooding in Cumbria December 2015. Secondary Flooding in Cumbria December 2015. https://www.metoffice.gov.uk/climate/uk/interesting/december2015. (Accessed July 2018).

22. Miller JD, Kjeldsen TR, Hannaford J, et al. A hydrological assessment of the November 2009 floods in Cumbria, UK. *Hydrology Research* 2013;44(1):180-97.

23. Department for Communities and Local Government. English indices of deprivation 2015. Secondary English indices of deprivation 2015. https://www.gov.uk/government/statistics/english-indices-of-deprivation-2015 (Accessed May 2018).

24. Kroenke K, Spitzer RL, Williams JB. The Patient Health Questionnaire-2: validity of a two-item depression screener. *Med Care* 2003;41(11):1284-92.

25. Kroenke K, Spitzer RL, Williams JB, et al. Anxiety disorders in primary care: prevalence, impairment, comorbidity, and detection. *Ann Intern Med* 2007;146(5):317-25.

26. Lang A, Stein M. An abbreviated PTSD checklist for use as a screening instrument in primary care. *Behaviour Research and Therapy* 2005;43(5):585-94.

27. van Reenen M, Janssen B. EQ-5D-5L User Guide. Version 2.1,2015:1-28. https://euroqol.org/wp-content/uploads/2016/09/EQ-5D-5L_UserGuide_2015.pdf (Accessed June 2018)

28. Department for Environment, Food and Rural Affairs. The National Adaptation Programme and the Third Strategy for Climate Adaptation Reporting. London, UK: Department for Environment, 2018. https://assets.publishing.service.gov.uk/government/uploads/system/uploads/attachment_data/file/727252/national-adaptation-programme-2018.pdf (Accessed September 2018).

29. Sayers PB, Horritt M, Penning Rowsell E, et al. Present and future flood vulnerability, risk and disadvantage: A UK scale assessment. A report for the Joseph Rowntree Foundation. Watlington, UK: Sayers and Partners LLP, 2017:1-98. http://www.sayersandpartners.co.uk/uploads/6/2/0/9/6209349/sayers_2017__present_and_future_flood_vulnerability_risk_and_disadvantage_-_final_report_-_uploaded_05june2017_printed_-_high_quality.pdf (Accessed September 2018).

For peer review only - http://bmjopen.bmj.com/site/about/guidelines.xhtml

30. Tan HZ, Luo YJ, Wen SW, et al. The effect of a disastrous flood on the quality of life in Dongting lake area in China. *Asia Pac J Public Health* 2004;16(2):126-32.

31. Reifels L, Mills K, Duckers MLA, O'Donnell ML. Psychiatric epidemiology and disaster exposure in Australia. *Epidemiol Psychiatr Sci* 2019;28(3):310-20.

32. Tapsell SM, Penning-Rowsell EC, Tunstall SM, et al. Vulnerability to flooding: health and social dimensions. *Philos Trans A Math Phys Eng Sci* 2002;360(1796):1511-25.

33. Tunstall S, Tapsell S, Green C, et al. The health effects of flooding: Social research results from England and Wales. *J Water Health* 2006;4(3):365-80.

34. Bulter C, Walker-Springett K, Adger WN, et al. Social and Political Dynamics of Flood Risk, Recovery and Response: A Report on the Findings of the Winter Floods Project. Exeter, UK: The University of Exeter, 2016:1-48. http://geography.exeter.ac.uk/media/universityofexeter/schoolofgeography/winterfloods/documents/Butler_et_al_2016_Socia_Political_DynamicsFloods.pdf (Accessed July 2018).

35. Knight BG, Gatz M, Heller K, Bengtson VL. Age and emotional response to the Northridge earthquake: a longitudinal analysis. *Psychol Aging* 2000;15(4):627-34.

36. Shrira A, Palgi Y, Hamama-Raz Y, Goodwin R, Ben-Ezra M. Previous exposure to the World Trade Center terrorist attack and posttraumatic symptoms among older adults following Hurricane Sandy. *Psychiatry* 2014;77(4):374-85.

37. Twigger-Ross C, Kashefi E, Weldon S, et al. Flood Resilience Community Pathfinder Evaluation - Rapid Evidence Assessment. London, UK: Department for Environment, Food and Rural Affairs, 2014. https://nationalfloodforum.org.uk/wp-content/uploads/2017/04/Flood-Resilience-Community-Pathfinder-Evaluation_Rapid-Evidence-Assessment.pdf (Accessed September 2018).

38. Lamond JE, Joseph RD, Proverbs DG. An exploration of factors affecting the long term psychological impact and deterioration of mental health in flooded households. *Environ Res* 2015;140:325-34.

39. Paranjothy S, Gallacher J, Amlot R, Rubin GJ, Page L, Baxter T, et al. Psychosocial impact of the summer 2007 floods in England. *BMC Public Health* 2011;11:145.

40. Devlin NJ, Brooks R. EQ-5D and the EuroQol Group: Past, Present and Future. *Appl Health Econ Health Policy* 2017;15(2):127-37.

41. Munro A, Kovats RS, Rubin GJ, et al. Effect of evacuation and displacement on the association between flooding and mental health outcomes: a cross-sectional analysis of UK survey data. *Lancet Planet Health* 2017;1(4):e134-e41.

For peer review only - http://bmjopen.bmj.com/site/about/guidelines.xhtml

Appendix I. Socio-demographic characteristics of participants according to exposure to flooding

Characteristic	All participants (n=531)*	Exposure group		
		Unaffected (n=90)	Disrupted (n=322)	Flooded (n=119)
**Median age (IQR)**	62.4 (49.1 to 72.1)	64.0 (53.9 to 76.8)	61.9 (46.7 to 71.6)	61.3 (48.3 to 71.2)
**Sex**				
Male	217 (41.2%)	55 (61.1%)	113 (35.5%)	49 (41.2%)
Female	310 (58.8%)	35 (38.9%)	205 (64.5%)	70 (58.8%)
**Ethnicity**				
White	523 (99.6%)	88 (100%)	316 (99.4%)	119 (100%)
Other	2 (0.4%)	0 (0%)	2 (0.6%)	0 (0%)
**Marital Status**				
Married/civil partners/cohabiting	340 (64.6%)	68 (76.4%)	196 (61.6%)	76 (63.9%)
Separated/divorced/widowed	127 (24.1%)	16 (18.0%)	80 (25.2%)	31 (26.1%)
Single/other	59 (11.2%)	5 (5.6%)	42 (13.2%)	12 (10.1%)
**Housing tenure**				
Owner/joint owner/family owned	433 (82.3%)	73 (82.0%)	262 (82.4%)	98 (82.4%)
Other	93 (17.7%)	16 (18.0%)	56 (17.6%)	21 (17.6%)
**Employment**				
Employed	252 (47.8%)	36 (40.4%)	155 (48.4%)	61 (51.7%)
Retired	238 (45.2%)	47 (52.8%)	144 (45.0%)	47 (39.8%)
Other	37 (7.0%)	6 (6.7%)	21 (6.6%)	10 (8.5%)
**Education level**				
Degree or above	235 (44.8%)	29 (32.6%)	153 (48.2%)	53 (44.9%)
Below degree level	172 (32.8%)	32 (36.0%)	102 (32.2%)	38 (32.2%)
Other	46 (8.8%)	8 (9.0%)	29 (9.2%)	9 (7.6%)
None (no formal qualifications)	71 (13.5%)	20 (22.5%)	33 (10.4%)	18 (15.3%)
**English deprivation quintile**				
1 (least deprived)	49 (9.4%)	17 (19.1%)	30 (9.4%)	2 (1.7%)
2	221 (42.2%)	38 (42.7%)	138 (43.4%)	45 (38.5%)
3	202 (38.6%)	25 (28.1%)	114 (35.9%)	63 (53.9%)
4 and 5**	52 (9.9%)	9 (10.1%)	36 (11.3%)	7(6.0%)
**Long-term illness**				
Yes	394 (76.5%)	69 (80.2%)	241 (77.0%)	84 (72.4%)
No	121 (23.5%)	17(19.8%)	72 (23.0%)	32 (27.6%)

Note: proportions calculated among those with known information on the characteristic of interest
*With known exposure status
**The two most deprived quintiles were combined due to small numbers

For peer review only

Appendix II. EQ-5D-5L health-related dimensions according to exposure to flooding

EQ-5D-5L dimension	Level	Exposure to flooding					
		All	Unaffected	Disrupted	Flooded (all)	Single flooded	Repeat flooded
		n (%)	n (%)	n (%)	n (%)	n (%)	n (%)
Mobility problems	No	391 (75.2%)	65 (73.9%)	240 (76.4%)	86 (74.6%)	41 (75.9%)	45 (70.3%)
	Slight	59 (11.3%)	11 (12.5%)	36 (11.5%)	11 (9.3%)	1 (1.9%)	10 (15.6%)
	Moderate	49 (9.4%)	8 (9.1%)	25 (8.0%)	16 (13.6%)	10 (18.5%)	6 (9.4%)
	Severe/unable*	22 (4.2%)	4 (4.5%)	13 (4.1%)	5 (4.2%)	2 (3.7%)	3 (4.7%)
Self-care problems	No	482 (92.7%)	84 (95.5%)	290 (93.0%)	108 (91.5%)	49 (90.7%)	59 (92.2%)
	Slight	18 (3.5%)	2 (2.3%)	13 (4.1%)	3 (2.5%)	1 (1.9%)	2 (3.1%)
	Moderate	16 (3.1%)	2 (2.3%)	8 (2.5%)	6 (5.1%)	4 (7.4%)	2 (3.1%)
	Severe/unable*	4 (0.8%)	0 (0%)	3 (1.0%)	1 (0.8%)	0 (0%)	1 (1.6%)
Activity problems	No	403 (77.5%)	71 (80.7%)	247 (78.7%)	85 (72.0%)	37 (68.5%)	48 (75.0%)
	Slight	55 (10.6%)	9 (10.2%)	32 (10.2%)	14 (11.9%)	7 (13.0%)	7 (10.9%)
	Moderate	48 (9.2%)	7 (8.0%)	28 (8.9%)	13 (11.0%)	8 (14.8%)	5 (7.8%)
	Severe/unable*	14 (2.7%)	1 (1.1%)	7 (2.2%)	6 (5.1%)	2 (3.7%)	4 (6.3%)
Pain/discomfort	No	311 (59.8%)	51 (58.0%)	194 (61.8%)	66 (55.9%)	29 (53.7%)	37 (57.8%)
	Slight	122 (23.5%)	25 (28.4%)	67 (21.3%)	30 (25.4%)	15 (28.8%)	15 (23.4%)
	Moderate	63 (12.1%)	8 (9.1%)	39 (12.4%)	16 (13.6%)	7 (13.0%)	9 (14.1%)
	Severe/extreme*	24 (4.6%)	4 (4.5%)	14 (4.5%)	6 (5.1%)	3 (5.6%)	3 (4.7%)
Anxiety/depression	No	347 (66.7%)	71 (80.7%)	232 (73.9%)	44 (37.3%)	19 (35.2%)	25 (39.1%)
	Slight	96 (18.5%)	13 (14.8%)	53 (16.9%)	30 (25.4%)	18 (33.3%)	12 (18.8%)
	Moderate	60 (11.5%)	3 (3.4%)	23 (7.3%)	34 (28.8%)	14 (25.9%)	20 (31.3%)
	Severe/extreme*	17 (3.3%)	1 (1.1%)	6 (1.9%)	10 (8.5%)	3 (5.6%)	7 (10.9%)

*Groups combined due to small numbers

For peer review only

STROBE Statement—Checklist of items that should be included in reports of ***cross-sectional studies***

	Item No	Recommendation
**Title and abstract**	1	(*a*) Indicate the study's design with a commonly used term in the title or the abstract – p.2
		(*b*) Provide in the abstract an informative and balanced summary of what was done and what was found – p.2
**Introduction**		
Background/rationale	2	Explain the scientific background and rationale for the investigation being reported - p.4
Objectives	3	State specific objectives, including any prespecified hypotheses – p.4
**Methods**		
Study design	4	Present key elements of study design early in the paper – p.5-6
Setting	5	Describe the setting, locations, and relevant dates, including periods of recruitment, exposure, follow-up, and data collection – p.5-6
Participants	6	(*a*) Give the eligibility criteria, and the sources and methods of selection of participants – p.5
Variables	7	Clearly define all outcomes, exposures, predictors, potential confounders, and effect modifiers. Give diagnostic criteria, if applicable – p.5-6
Data sources/ measurement	8*	For each variable of interest, give sources of data and details of methods of assessment (measurement). Describe comparability of assessment methods if there is more than one group – p.5-6
Bias	9	Describe any efforts to address potential sources of bias – p.5
Study size	10	Explain how the study size was arrived at – p.5
Quantitative variables	11	Explain how quantitative variables were handled in the analyses. If applicable, describe which groupings were chosen and why – p.5-6
Statistical methods	12	(*a*) Describe all statistical methods, including those used to control for confounding – p.6
		(*b*) Describe any methods used to examine subgroups and interactions – p.6
		(*c*) Explain how missing data were addressed – p.6
		(*d*) If applicable, describe analytical methods taking account of sampling strategy N/A
		(*e*) Describe any sensitivity analyses – N/A
**Results**		
Participants	13*	(a) Report numbers of individuals at each stage of study—eg numbers potentially eligible, examined for eligibility, confirmed eligible, included in the study, completing follow-up, and analysed – p.6-7
		(b) Give reasons for non-participation at each stage – N/A
		(c) Consider use of a flow diagram – not necessary
Descriptive data	14*	(a) Give characteristics of study participants (eg demographic, clinical, social) and information on exposures and potential confounders – p.6-7 and Appendix I
		(b) Indicate number of participants with missing data for each variable of interest – p.6-7
Outcome data	15*	Report numbers of outcome events or summary measures – p.6-7
Main results	16	(*a*) Give unadjusted estimates and, if applicable, confounder-adjusted estimates and their precision (eg, 95% confidence interval). Make clear which confounders were adjusted for and why they were included – p.7 and Tables 1, 2, 3 and 4

For peer review only - http://bmjopen.bmj.com/site/about/guidelines.xhtml

		(*b*) Report category boundaries when continuous variables were categorized – p.5
		(*c*) If relevant, consider translating estimates of relative risk into absolute risk for a meaningful time period – N/A
Other analyses	17	Report other analyses done—eg analyses of subgroups and interactions, and sensitivity analyses – N/A
**Discussion**		
Key results	18	Summarise key results with reference to study objectives – p.8
Limitations	19	Discuss limitations of the study, taking into account sources of potential bias or imprecision. Discuss both direction and magnitude of any potential bias – p.8-9
Interpretation	20	Give a cautious overall interpretation of results considering objectives, limitations, multiplicity of analyses, results from similar studies, and other relevant evidence – p8-9
Generalisability	21	Discuss the generalisability (external validity) of the study results – p.8-9
**Other information**		
Funding	22	Give the source of funding and the role of the funders for the present study and, if applicable, for the original study on which the present article is based – p.11

*Give information separately for exposed and unexposed groups.

Note: An Explanation and Elaboration article discusses each checklist item and gives methodological background and published examples of transparent reporting. The STROBE checklist is best used in conjunction with this article (freely available on the Web sites of PLoS Medicine at http://www.plosmedicine.org/, Annals of Internal Medicine at http://www.annals.org/, and Epidemiology at http://www.epidem.com/). Information on the STROBE Initiative is available at www.strobe-statement.org.

For peer review only
